# Synthesis of biologically active Shiga toxins in cell-free systems

**DOI:** 10.1038/s41598-024-56190-3

**Published:** 2024-03-13

**Authors:** Franziska Ramm, Danny Kaser, Irina König, Juliane Fellendorf, Dana Wenzel, Anne Zemella, Panagiotis Papatheodorou, Holger Barth, Herbert Schmidt

**Affiliations:** 1 Fraunhofer Institute for Cell Therapy and Immunology, Branch Bioanalytics and Bioprocesses (IZI-BB), Am Mühlenberg 13, 14476 Potsdam, Germany; 2 Institute of Nutritional Science – Nutritional Toxicology, University of Potsdam, Arthur-Scheunert-Allee 114-116, 14558 Nuthetal, Germany; 3 Institute of Experimental and Clinical Pharmacology, Toxicology and Pharmacology of Natural Products, Ulm University Medical Center, Albert-Einstein-Allee 11, 89081 Ulm, Germany; 4 Department of Food Microbiology and Hygiene, Institute of Food Science and Biotechnology, University of Hohenheim, Garbenstraße 28, 70599 Stuttgart, Germany

**Keywords:** Proteins, Expression systems, Bacterial toxins, Assay systems

## Abstract

Shiga toxins (Stx) produced by pathogenic bacteria can cause mild to severe diseases in humans. Thus, the analysis of such toxins is of utmost importance. As an AB_5_ toxin, Stx consist of a catalytic A-subunit acting as a ribosome-inactivating protein (RIP) and a B-pentamer binding domain. In this study we synthesized the subunits and holotoxins from Stx and Stx2a using different cell-free systems, namely an *E. coli-* and CHO-based cell-free protein synthesis (CFPS) system. The functional activity of the protein toxins was analyzed in two ways. First, activity of the A-subunits was assessed using an in vitro protein inhibition assay. StxA produced in an *E. coli* cell-free system showed significant RIP activity at concentrations of 0.02 nM, whereas toxins synthesized in a CHO cell-free system revealed significant activity at concentrations of 0.2 nM. Cell-free synthesized StxA2a was compared to StxA2a expressed in *E. coli* cells. Cell-based StxA2a had to be added at concentrations of 20 to 200 nM to yield a significant RIP activity. Furthermore, holotoxin analysis on cultured HeLa cells using an O-propargyl-puromycin assay showed significant protein translation reduction at concentrations of 10 nM and 5 nM for cell-free synthesized toxins derived from *E. coli* and CHO systems, respectively. Overall, these results show that Stx can be synthesized using different cell-free systems while remaining functionally active. In addition, we were able to use CFPS to assess the activity of different Stx variants which can further be used for RIPs in general.

## Introduction

Until today infections with pathogenic bacteria remain a major threat to human and animal health. Many pathogenic bacteria produce highly toxic proteins that efficiently enter mammalian cells via a binding/transport -subunit and act as enzymes in their cytosol. The toxin-catalyzed substrate modification leads to characteristic cellular reactions and thus results in the typical clinical symptoms associated with the individual toxin and toxin-producing bacteria^1,2^. A major class of protein toxins derived from pathogenic bacteria are AB_5_ toxins. These toxins reflect a characteristic multicomponent structure of a catalytic A-subunit and a pentameric ring structure of the B-subunit, necessary for cell binding^3^. Both subunits together form the complete holotoxin.

Shiga toxin (Stx) is a prominent example for an AB_5_ toxin. In humans, Stx can cause mild tissue symptoms such as watery diarrhea but also leads to severe disease patterns such as the hemolytic uremic syndrome. Originally, Stx derived from *Shigella dysenteriae* ^4^. During the last decades, relatives of Stx have been isolated in *Escherichia coli* strains and are classified as Stx1 and Stx2^3,5, 6^. Stx2a is mainly found in *E. coli* O157:H7 and currently the most common representative^7,8^. Stx1 and Stx2 are only 60% homologous in their amino acid sequences^3^, which underlines the necessity to study the activities of the individual toxins. Nonetheless, the general mode of action of the individual Stx is similar. The catalytic A-subunit possesses an rRNA *N*-glycosidase activity and targets eukaryotic ribosomes at the glycosidic bond of the adenine base A_4324_ / A_4326_ of the sarcin-ricin-loop of the 28S rRNA of the 60S ribosomal subunit^9,10^. This ultimately leads to the inhibition of protein synthesis within the target cell and eventually to cell death^11,12^. Toxins with such a molecular mode of action are also referred to as ribosome-inactivating proteins (RIPs). The StxA-subunit has a molecular weight of about 30–32 kDa and consists of two domains, the A1- and A2-subunit, which are defined by a furin and trypsin sensitive region. The protein is nicked but both subunits are further connected by a disulfide bond. The A1-subunit carries the catalytic domain. The A2-subunit builds an anchor between the A1-subunit and the pentameric B-ring^6,13^. The StxB-subunit is a small protein of approximately 8 kDa and forms a cell binding pentameric complex. Studies have shown that each B-subunit has up to three receptor binding sites to the globotriaosylceramide; Gb_3_ (CD77) receptor^14,15^. The StxB-subunit shows the highest binding efficiencies to Gb_3_, but studies have identified that cells presenting Gb_4_ and colonic cells without these receptors were able to bind to StxB^16,17^. The exact cell binding of Stx still remains to be elucidated as other studies have also found that the binding does not only depend on the glycan-structure but rather on the lipid environment^18^. After cell binding by the B-subunit, the pathogenesis is facilitated by clathrin-dependent uptake and retrograde trafficking to the Golgi apparatus und further to the Endoplasmic Reticulum (ER). In the ER the A-subunit is split into the A1 and A2 domain, which triggers the catalytic activity of the A1 domain. After its release to the cytoplasm, the A1-subunit targets eukaryotic ribosomes^3,19^. This indicates a high need for novel techniques to study individual toxin variants as well as cellular pathways.

In order to efficiently study the cellular uptake and molecular mode of action of bacterial protein toxins in more detail, the availability of functional toxins and individual toxin subunits is mandatory. The generation of synthetically synthesized toxins is needed, especially if standardized functionality assessments are required. Standard procedures in bioproduction focus on protein synthesis based on pro- and eukaryotic cell-based systems^20,21^. Unfortunately, the nature of the toxins themselves limits the expression of the toxin as the producing cells are damaged. Thus, many studies focus on the expression of truncated or inactivated toxin fragments^22,23^. Further, cellular expression of toxins can lead to higher safety regulations as genetically modified organisms might be generated^24^. An alternative to such cell-based expression approaches is cell-free protein synthesis (CFPS). As a cell lysate rather than viable cells is used, the viability of the expression system does not have to be maintained and no high laboratory standards are required. No cellular barriers limit the reaction and thus an open system is available that can easily be adapted to the needs of the protein of interest^25,26^. A further opportunity of cell-free systems, in particular eukaryotic systems, is the endotoxin-free environment of the system which allows for a direct use of the protein in downstream applications without any need of additional purification steps. The scalability of the system offers protein synthesis in small scales (e.g. microwell plates) allowing for rapid screening of different proteins or reaction modes^25^. These advantages qualify CFPS for the synthesis and analysis of toxic proteins^27–29^.

Within this study, the functionality of Stx and Stx2a originating from *S. dysenteriae* and *E. coli* O157:H7, respectively, were analyzed in terms of enzyme activity of the A-subunit in vitro and in terms of biologic activity in cell intoxication. These variants were chosen to assess functional differences between toxins. The synthesis of both toxin variants was performed using two cell-free systems: the prokaryotic *E. coli* system^30^ as well as a eukaryotic system based on Chinese hamster ovary (CHO) cells^31^ as these systems are well-established cell-free systems. As Stx harms eukaryotic ribosomes, an optimized eukaryotic system harboring microsomal vesicles derived from the endoplasmic reticulum (ER) was used^32^. The signal sequence of the honey bee peptide melittin (Mel) was fused in frame to the protein-coding sequence to translocate the synthesized Stx into these microsomal vesicles co-translationally and allow for protein synthesis^32^. Both cell-free systems were compared to StxA2a that could be produced in cell-based *E. coli* expression.

The synthesis of both toxin variants was established and the functionality assessed using in vitro and cell-based protein inhibition assays. It was shown that both variants were functionally active, but the Stx variant showed a higher toxicity. In addition, both cell-free systems were qualified to synthesize functionally active toxins. To our knowledge, this is the first study to assess the functionality of two Stx toxins in parallel, while also comparing different expression systems. Both cell-free systems showed functionally active Stx proteins at lower concentrations as compared to cell-derived StxA2a, hence demonstrating the use of CFPS as a fast and efficient method to study proteinaceous toxins.

## Results

### Cell-free synthesis of Stx and Stx2a

**Figure 1:**
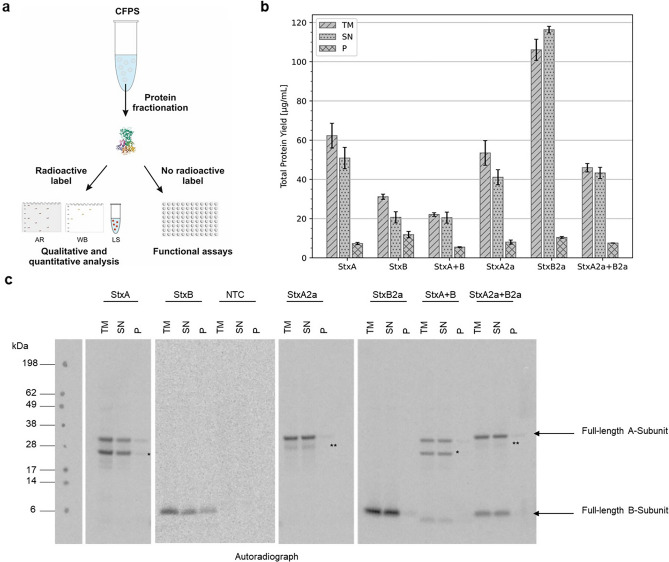
CFPS of Stx in an *E. coli* system. (**a**) Schematic overview of CFPS pipeline starting from synthesis to the analysis including autoradiography (AR), Western blotting (WB) and liquid scintillation (LS). Protein structure derived from PDB 1R4Q^33^. Stx single subunits were synthesized in *E. coli* lysate either separately or in a co-expression (1:5 molar plasmid ratio, StxA + StxB and StxA2a + StxB2a). Samples were fractionated and the crude translation mixture (TM), the soluble supernatant fraction (SN) and the accumulated protein pellet (P). (**b**) Quantitative analysis of cell‐free synthesized Stx subunits as performed by liquid scintillation counting. Standard deviations were calculated from triplicate analysis. (**c**) Autoradiograph showing ^14^C‐leucine labeled Stx protein subunits. The NTC as a lysate background control was analyzed in the same manner as the protein samples and each fraction TM, SN and P were visualized in the autoradiograph. Cleavage Product of StxA and StxA2a were marked with * and **, respectively.

Until today, Stx has not been produced in a cell-free manner and thus the synthesis of the Stx toxin subunits was established in two cell-free systems. In general, the synthesis pipelines in both systems were similar. Proteins were radioactively labeled with ^14^C-leucine, fractionated and subsequently qualitatively and quantitatively analyzed. Non-labeled proteins were directly used for functionality assays (Fig. 1a).

#### Prokaryotic *E. coli* cell-free system

As an initial analysis, the synthesis of Stx subunits was established using an *E. coli* cell-free system. Quantitative analysis indicated that all proteins could be synthesized and were mainly found in the supernatant (SN) as soluble proteins rather than in the pelleted debris of the lysate (P). In general, the co-expression of both subunits to form the holotoxin resulted in lower total protein yields as compared to the expression of the single subunits. Specifically, the Stx holotoxin yielded a total of 22.06 µg/mL with a soluble fraction of 20.51 µg/mL. The Stx2a holotoxin was synthesized in a soluble fraction of 43.30 µg/mL. While the yields of the A-subunit of both variants was fairly similar, the StxB2a-subunit from *E. coli* could be synthesized in a higher amount than StxB originating from *S. dysenteriae* (Fig. 1b). Autoradiography of the synthesized proteins depicted intense proteins band for every individual subunit as well as the co-expressed subunits of the holotoxins in the translation mixture (TM) and in the SN but not in the P fraction. Thus, the qualitative results are compatible with the quantitative results. A no template control (NTC) was used as a lysate background and did not show any distinct band in neither fraction. Further, the A-subunit of both variants showed a cleavage product of the whole A-subunit (Fig. 1c, uncropped autoradiographs in Supplementary Fig. 1).

#### Eukaryotic CHO cell-free system

**Figure 2:**
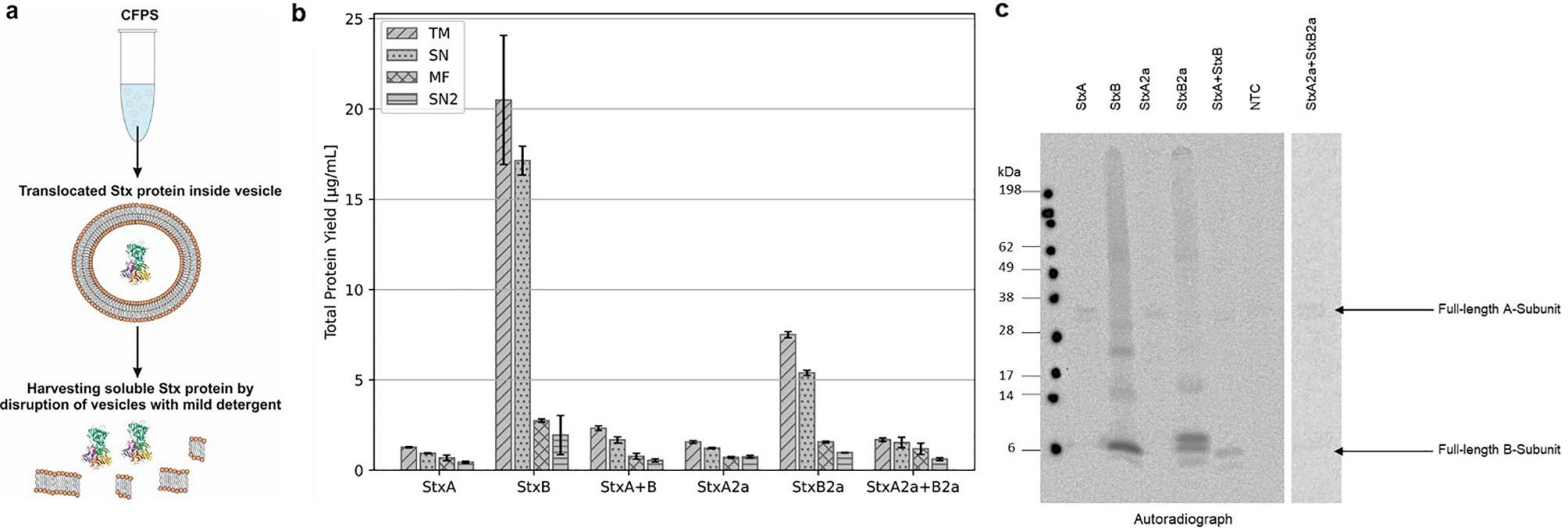
CFPS of Stx in a CHO-based system. (**a**) Schematic overview of the co-translational translocation of Stx subunits. Protein structure derived from PDB 1R4Q^33^. Stx single subunits were synthesized in a CHO lysate either separately or in a co-expression (1:5 molar plasmid ratio, StxA + StxB and StxA2a + StxB2a). (**b**) Quantitative analysis of synthesized Stx subunits and co-expressed holotoxins by liquid scintillation counting. Standard deviations were calculated from triplicate analysis. (**c**) Qualitative analysis by autoradiography showing ^14^C‐leucine labeled Stx proteins. The NTC as a lysate background control was analyzed in the same manner as the protein samples visualized in the autoradiograph.

The synthesis of a RIP that specifically targets eukaryotic ribosomes within a eukaryotic CHO expression system is challenging. Hence, the toxins were expressed as fusion proteins with a Mel signal peptide to co-translationally translocate the newly synthesized Stx proteins into the ER-based vesicles, thus encapsulating Stx, therefore not being able to target the ribosomes. After the synthesis the proteins were harvested from microsomal fraction (MF) using a mild detergent resulting in soluble and active proteins from the second supernatant (SN2) (Fig. 2a). Quantitative analysis showed that the overall total protein yields were decreased in comparison to the *E. coli*-based system. Unfortunately, most soluble protein was detected in the SN fraction and not in the SN2 fraction. This indicated that the co-translational translocation using the Mel signal peptide was not fully accomplished. Further, it could be identified that the synthesis rate of the StxA-subunits was lower than that of the StxB-subunits, even though the Mel signal peptide was used. The total protein yield of the holotoxins was calculated at 2.32 and 1.68 µg/mL for the Stx and Stx2a holotoxin, respectively (Fig. 2b). These findings of a low StxA protein yield further indicate an insufficient co-translational translocation as the active A-subunit might have inhibited its own cell-free synthesis in the eukaryotic CHO system. Interestingly, StxB2a was produced in lower amounts than StxB (Fig. 2b). Nonetheless, autoradiography confirmed that proteins could be synthesized in a eukaryotic cell-free manner, as all protein bands were visible (Fig. 2c, uncropped autoradiographs in Supplementary Fig. 2).

#### Stx subunit analysis

**Figure 3:**
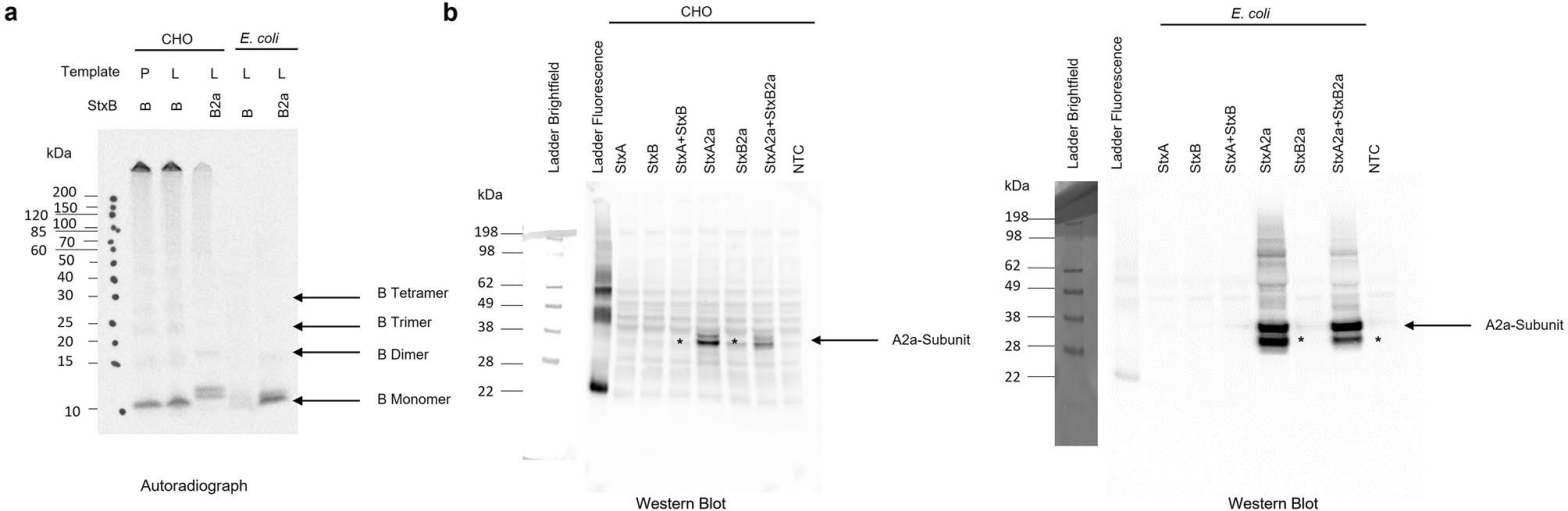
Stx subunit analysis. (**a**) Qualitative analysis by autoradiography showing ^14^C‐leucine labeled StxB and StxB2a proteins after a 48 h CECF synthesis reaction in CHO and *E. coli* lysate and potential multimers. P equals the plasmid DNA template and L equals the linear DNA template. (**b**) Qualitative analysis by western blotting using the Shiga toxin 2 antibody 11E10. Stx single subunits were synthesized in a CHO (left) or *E. coli* (right) lysate either separately or in a co-expression (1:5 molar plasmid ratio, StxA + StxB and StxA2a + StxB2a). Cleavage Product of StxA2a was marked with *.

In a next step, the multimerization of the B-subunits was assessed as the pentameric ring formation is considered an essential part of the cell binding process. To further increase the total protein yield of the synthesized proteins to enhance complex formation, a continuous exchange cell-free reaction (a so-called dialysis mode) was used^32,34^. In a first attempt, both, StxB- and StxB2a-subunits were synthesized in a CECF reaction for 24 and 48 h using both cell-free systems. The multimerization was assessed in the SDS-PAGE under non-reducing and reducing (addition of 50 mM Dithiothreitol (DTT)) conditions. After 48 h the qualitative analysis of the autoradiograph showed that StxB based on plasmid template could be synthesized more efficiently than StxB2a based on linearized templates. The synthesis efficiency of the CHO system was higher than that of the E. coli system. In the CHO-based system a multimerization could be detected in reducing and non-reducing conditions (Supplementary Fig. 3). The multimerization was independent of the synthesis time in the CHO-based system. As the difference in the protein amount might have been caused by a lower amount of DNA template present in the reaction due to different templates (CHO-system: StxB plasmid DNA, StxB2a linear DNA template, *E. coli*: both variants linear DNA template). Thus, in a next step, a CECF reaction was performed for 48 h, subsequently the total protein yields of the individual samples were assessed and 100 ng of each individual protein were loaded onto the SDS-PAGE. These data indicate that the CHO system efficiently multimerizes both variant B-subunits while the *E. coli* system does not (Fig. 3).

The detection of proteins is relevant for diverse applications such as diagnostic tools or cell binding assays. Therefore, a Western Blot was performed to identify the binding of Stx antibodies against cell-free synthesized proteins. Stx single subunits and co-expressed holotoxins from both variants (Stx and Stx2a) were synthesized in a cell-free manner using both lysates. The Shiga toxin 2 antibody 11E10 detected the StxA2a-subunit when expressed individually or when co-expressed with the StxB2a-subunit. The StxA-subunit was neither detected when expressed in the CHO nor when expressed in the *E. coli* lysate (Fig. 3b). As this antibody is recommended for the detection of Stx2 toxins derived from *E. coli* strains, these results further identify this antibody as an Stx2 specific antibody. Autoradiography of the blots identified respective protein band and thus confirmed the successful synthesis and detection of the Stx proteins (Supplementary Fig. 4). The 11E10 antibody is known to detect the A1 subunit of Stx2 and putatively binds three epitopes that are different from Stx1^35^. To confirm our findings of the Western Blot, we isolated both protein bands from CHO and *E. coli* cell-free synthesized StxA and StxA2a samples from SDS-PAGEs and analyzed them using mass spectrometry (MS). These data showed that the CHO samples clearly included the presence of the StxA and StxA2a proteins within the individual samples (Supplementary Table 1). Samples synthesized in *E. coli* lysate showed StxA within all samples indicating that the gels were overloaded and impurities across samples occurred (Supplementary Table 2). As the cleavage product within the StxA2a sample was majorly present in the autoradiograph and Western Blot after an *E. coli* synthesis, we further analyzed the MS data and could confirm that the three putative epitopes of the StxA2a region postulated by Smith et al. (residues 42 to 49, residues 96 to 100 and residues 244 to 259) were present in both protein bands of the cell-free synthesized StxA2a samples (Supplementary Fig. 5).

All in all, these data showed that both cell-free systems can be efficiently used for the synthesis and analysis of Stx subunits from different Stx variants.

### In vitro protein inhibition assay

In order to identify an expression system as suitable, the functionality of the respective protein of interest has to be analyzed. Therefore, an in vitro protein inhibition assay using the model protein Luciferase (Luc) was performed to validate the functionality of the catalytic A-subunit of the Stx proteins. The total protein yield of Luc, the qualitative analysis as well as a Luc activity assay in both eukaryotic CHO system and prokaryotic *E. coli* system were used to identify whether the StxA- and StxA2a-subunits were functionally active. Further, this assay was used to detect differences in the activity of Stx and Stx2a. Control reactions (without toxin addition) exhibit a high Luc protein yield and activity.

#### Activity of Stx after prokaryotic *E. coli* cell-free synthesis

In an initial test, StxA and StxA2a were synthesized in an *E. coli* cell-free manner and added to the Luc synthesis in the CHO and *E. coli* cell-free systems afterwards. These data indicated that 2 nM and even 0.2 nM of both Stx efficiently reduced the amount of total Luc as well as the activity in the CHO-based synthesis but not in the *E. coli*-based synthesis (Supplementary Fig. 6).

**Figure 4:**
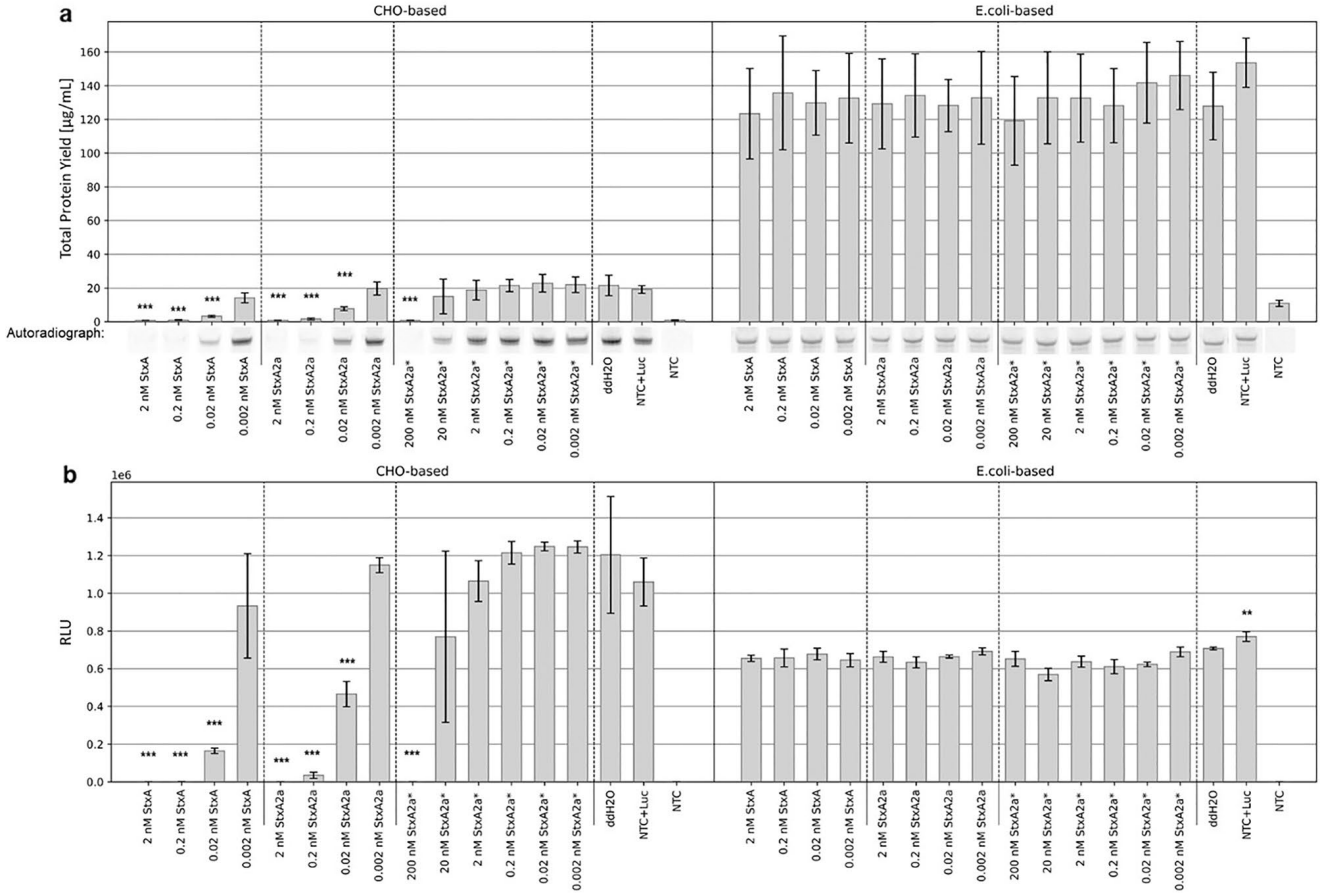
In vitro protein inhibition with Stx variants from an *E. coli* cell-free system. Luc was synthesized in an *E. coli* cell-free system. The addition of water or NTC was monitored as negative controls and StxA variants (StxA and StxA2a) at a concentration range of 0.002 to 2 nM as well as the positive control, StxA2a from cell-based *E. coli* synthesis (StxA2a*) were added to assess the protein synthesis inhibition ability. (**a**) Quantitative analysis of synthesized Luc by liquid scintillation counting. Standard deviation derived from two assays with triplicate analysis (n = 6). Statistical significance by ANOVA according to Bonferroni and Tuckey as indicated by * as compared to Luc + ddH_2_O. Qualitative analysis by autoradiography showing ^14^C‐leucine labeled Luc. (**b**) Luc activity as measured by relative light units (RLU). Standard deviation derived from two assays with duplicate analysis (n = 4). Statistical significance by ANOVA according to Bonferroni and Tuckey as indicated by * as compared to Luc + ddH_2_O.

To validate these data, the experiment was reproduced and an additional control, StxA2a from a cell-based *E. coli* synthesis (termed as StxA2a*), was implemented in the following experiments. *E. coli*-based cell-free synthesized Stx proteins were supplemented in a concentration range from 0.002 nM to 2 nM. The StxA2a* control was further supplemented at 200 and 20 nM (Supplementary Fig. 7). All data were compared to the negative controls, namely a regular Luc synthesis without the addition of a supplement (Luc + ddH_2_O) and Luc with the addition of a NTC volume equivalent (Luc + NTC). The Luc + NTC samples served as a background control to assess the effect of the components of the cell-free synthesis on the Luc synthesis itself. The total protein yield of the Luc was steady over all samples in the *E. coli*-based system, but a statistically significant decreased protein yield compared to Luc + ddH_2_O could be detected when 0.02 to 2 nM cell-free synthesized and 200 nM cell-based Stx variants were added to the CHO-based synthesis of Luc (Fig. 4a). These results correlate with the qualitative analysis of the autoradiographs as demonstrated by less intense protein bands (Fig. 4a, complete autoradiography Supplementary Fig. 8a). At last, the functional activity of the Luc was assessed. Luc that was synthesized in a CHO cell-free system showed a statistically significant reduced activity when 0.02 to 2 nM cell-free synthesized and 200 nM cell-based Stx variants were added. No influence of Stx variants upon the *E. coli* synthesized Luc could be detected (Fig. 4b).

All in all, these data show that already low concentrations of about 0.02 nM cell-free synthesized Stx protein are functionally active and significantly inactivate eukaryotic ribosomes, demonstrating the effectiveness of the cell-free system.

#### Activity of Stx after eukaryotic CHO cell-free synthesis

The data gathered for Stx synthesized in a prokaryotic cell-free system already indicated the ability of the cell-free system to synthesize a RIP. Hence, it was assessed whether the eukaryotic cell-free system and the strategy of using a Mel signal peptide for translocation into the microsomal vesicles was also suitable to synthesize a functionally active RIP.

**Figure 5:**
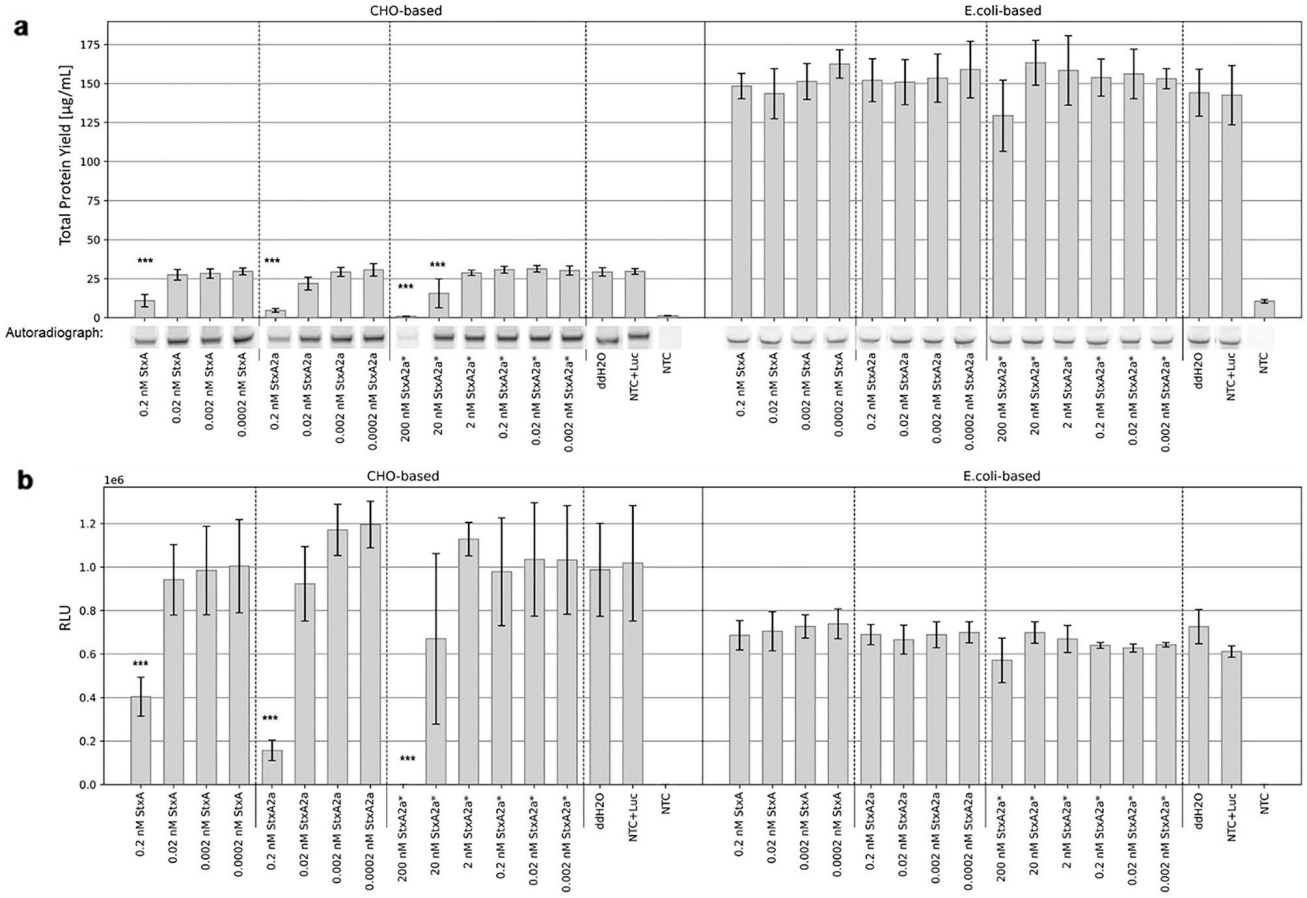
In vitro protein inhibition with Stx variants from a CHO cell-free system. Luc was synthesized in a CHO cell-free system. The addition of water or NTC was monitored as negative controls and StxA variants (StxA and StxA2a) at a concentration range of 0.0002 to 0.2 nM as well as the positive control, StxA2a from cell-based *E. coli* synthesis (StxA2a*) were added to assess the protein synthesis inhibition ability. a) Quantitative analysis of Luc by liquid scintillation counting. Standard deviation was calculated from two assays with triplicate analysis (n = 6). Statistical significance by ANOVA according to Bonferroni and Tuckey as indicated by * as compared to Luc + ddH_2_O. b) Qualitative analysis by autoradiography showing ^14^C‐leucine labeled Luc. c) Luc activity measured by relative light units (RLU). Standard deviation calculated from two assays with duplicate analysis (n = 4). Statistical significance by ANOVA according to Bonferroni and Tuckey as indicated by * as compared to Luc + ddH_2_O.

The identical experimental set up was chosen to validate the system. The only difference was that StxA and StxA2a were synthesized in CHO-based cell-free manner and subsequently added to the synthesis of the model protein Luc. The CHO-based synthesized proteins were added in a concentration range of 0.0002 to 0.2 nM. All samples were compared to the general Luc synthesis (Luc + ddH2O) and the NTC control (Luc + NTC). Again, the Luc + NTC sample served as a background control. The overall total protein yield of the Luc after an *E. coli*-based synthesis was comparable between all samples. After Luc was synthesized in a CHO cell-free system, the addition of 0.2 nM StxA and StxA2a as well as 200 and 20 nM StxA2a* resulted in significantly reduced protein yields of Luc (Fig. 5a). In the CHO-based Luc synthesis system, autoradiography again confirmed the quantitative data from liquid scintillation counting and depicted less intense protein bands for Luc synthesis when 0.2 nM StxA2a and 200 nM StxA2a* were added. A minor reduction in the intensity of the protein band can be seen for samples where Luc was synthesized in the presence of 0.2 nM StxA and 20 nM StxA2a*. No intensity changes of protein bands were detected for Luc protein bands after an *E. coli* synthesis. (Fig. 5a, complete autoradiographs Supplementary Fig. 8b). The assessment of the functional activity of Luc indicated that upon the addition of 0.2 nM StxA2a and 200 nM StxA2a* led to a statistically significant reduction of activity in the CHO synthesis. The Luc activity after an *E. coli*-based synthesis remained steady (Fig. 5b).

All in all, these data show that the CHO cell-free system was also capable to synthesize functionally active Stx proteins. Interestingly, Stx proteins synthesized in both *E. coli* and CHO cell-free systems appeared to be more active than cell-based Stx samples. As the cell- based Stx samples were stored and cell-free synthesized proteins were synthesized freshly, a further experiment was performed with cell-free synthesized proteins that were stored at − 80 °C for the same time as the cell-based samples. These data indicated that the storage did not lead to the differences in the activity (Supplementary Figs. 9 and 10).

### Cell-based protein inhibition assay

**Figure 6:**
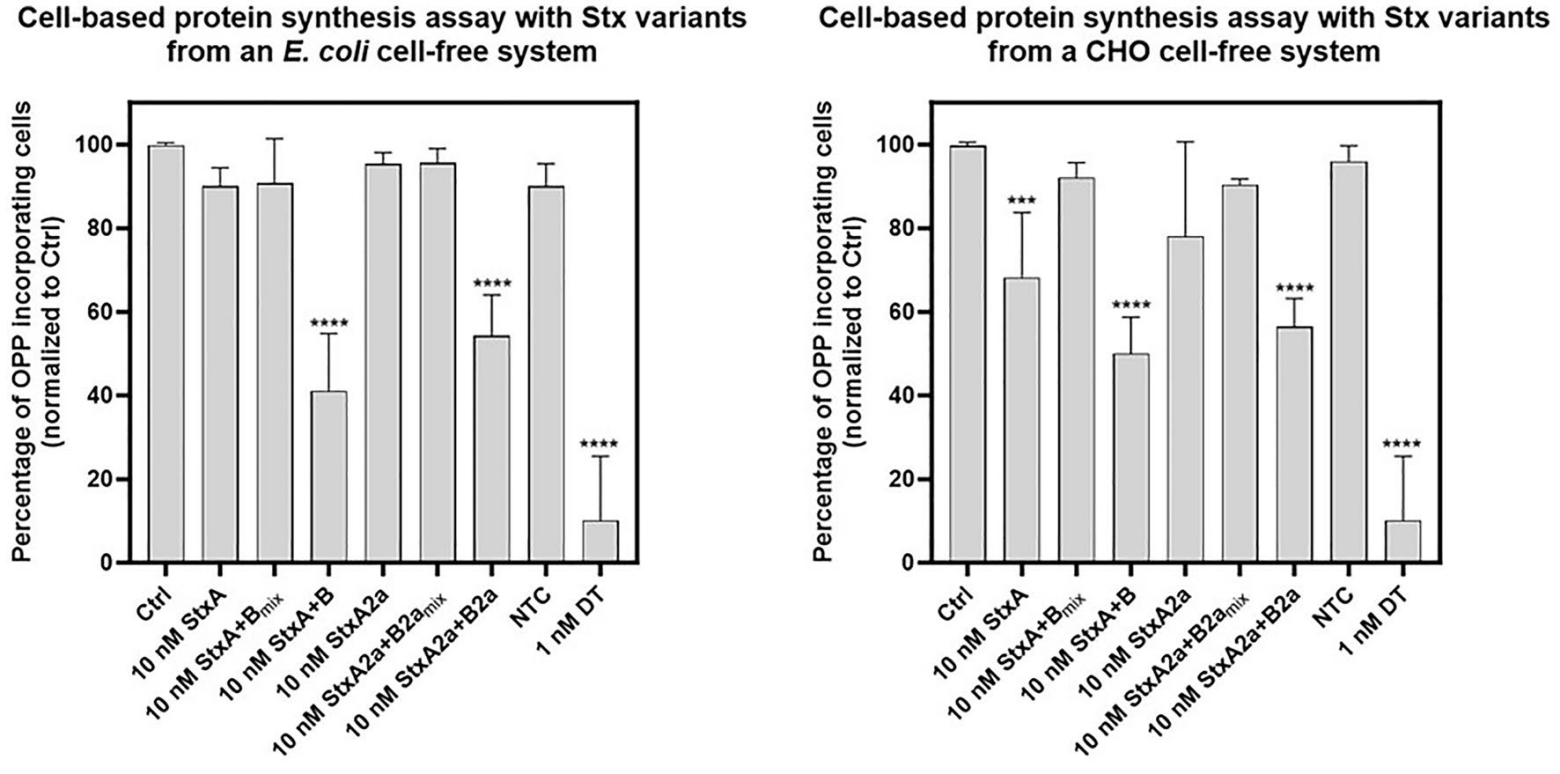
Quantitative analysis of OPP-based detection of protein synthesis in HeLa cells intoxicated with Stx variants from a *E. coli* or CHO cell-free system. HeLa cells were intoxicated with Stx variants for 18 h in a concentration of 10 nM (Stx variants from *E. coli* lysate) or 5 nM (Stx variants from CHO lysate). For this purpose, the cells were either treated with the A-subunits (StxA, StxA2a) on their own or with a combination of the A- and B-subunits in a molar ratio of 1:5 (StxA + B_mix_, StxA2a + B2a_mix_). In addition, cells were treated with the Stx variants where the A- and B-subunits were co-expressed in a molar ratio of 1:5 (StxA + B, StxA2a + B2a). Untreated cells (Ctrl) and cells incubated with NTC served as negative controls. As a positive control, cells were intoxicated with 1 nM diphtheria toxin (DT), which is a known inhibitor of protein synthesis. Shown is the percentage of OPP-incorporating cells relative to control (Ctrl), which was set to 100% and is represented by cells with maximal OPP incorporation as measure of protein biosynthesis. For the left graph values are given as mean ± SD of three independent assays with triplicates (n = 9; except for NTC (n = 8) and Stx(A + B) (n = 7)). Values from right graph are given as mean ± SD of two independent assays with triplicates (n = 6; except for NTC (n = 5). Statistical significance was determined by one-way ANOVA in combination with Dunnett’s correction for multiple comparison.

Finally, we aimed to verify that the Stx proteins synthesized in cell-free systems were able to enter into cells and to inhibit protein biosynthesis. For that purpose, an O-propargyl-puromycin (OPP)-based protein synthesis assay and HeLa cells were used analyzed by FACS. This is a direct measure for OPP incorporation and consequently of protein biosynthesis. Therefore, control cells (without toxin addition) exhibit a high fluorescence, but in cells treated with toxins that inhibit protein biosynthesis, the fluorescence is decreased. We found that co-expressed StxA and StxB as well as co-expressed StxA2a and StxB2a, either synthesized in *E. coli* or CHO cell-free systems, were both lowering the percentage of OPP incorporating cells, indicative of inhibition of protein biosynthesis (Fig. 6). Diphtheria toxin (DT) served as positive control for a known toxin that strongly inhibits protein biosynthesis. The single components alone and lysates from the no template controls (NTC, background conrol) were not significantly decreasing protein biosynthesis in HeLa cells, with the exception of StxA expressed in the CHO cell-free system. It has been shown that StxA can enter into target cells also independent of the B-subunit^36^. It seems that StxA expressed in the CHO cell-free system adopts a conformation that enables efficient binding and uptake into HeLa cells. Surprisingly, co-addition of independently expressed A- and B-subunits, either from *E. coli* or CHO cell-free systems, did not inhibit protein synthesis in HeLa cells.

## Discussion

Stx toxins cause an estimated case number of almost 3 million illnesses per year with a range of symptoms^37–39^. Depending on the EHEC strain and respective toxin that is produced, the disease severity caused after an intoxication can vary^7,38^. Thus, it is of utmost importance that individual variants und subtypes of Stx can be analyzed in a fast and efficient manner.

This study presents an approach to assess the functional activity of different Stx in diverse parallelizable and scalable methodological approaches. Traditional laboratory expression of Shiga toxins is performed in prokaryotic cell-based systems where the toxins are isolated from the strain^40–43^. Some studies even investigated different *stx* gene expressions in bacterial strains or directly performed infection studies with the bacterial strain^42,44^. Hence, a prokaryotic cell-free system was used to compare our findings to standard methods. Stx are ribosome inactivating proteins (RIPs) and the A-subunit specifically inactivates eukaryotic ribosomes. Therefore, the synthesis of the A-subunit is difficult in eukaryotic systems, but, the synthesis of complex RIPs which need posttranslational modification might require eukaryotic synthesis systems. Therefore, this study used a eukaryotic cell-free system by adapting the DNA template with a Mel signal peptide This leads to the translocation of the RIP into the ER-based microsomal vesicles^32^ and thus can no longer attack the ribosomes that are located outside of the microsomes. In prior work, the Mel signal peptide was shown to be very efficient in eukaryotic cell-free systems^45–47^ and the resulting translocation into the vesicles even protects from protease degradation^48^. As the data presented here show, the total protein yield of the synthesized A-subunit of the Stx variants was comparatively low, especially in comparison to the prokaryotic *E. coli*-based system (see Fig. 1 and 2). This indicates that the synthesis of the A-subunit is possible but self-limiting in a eukaryotic cell-free system. Some studies focusing on the B-subunit have shown that the B-subunit and modified variants of it can be synthesized in eukaryotic systems such as tobacco cells^49^ which correlates to the results in this study, showing a higher synthesis rate of the B-subunit in eukaryotic systems. Using quantitative western blotting, a study by Pellino et al. showed that Stx1 subunits expressed and purified from *E. coli* strains resulted in lower yields (StxA 1.9 µg/mL, StxB 1.3 µg/mL) as compared to Stx2a-subunits (StxA 4.6 µg/mL, StxB 9.1 µg/mL)^50^. A further study showed that Stx1 recombinantly expressed from *E. coli* cells could be purified with a yield of 2–3 mg/L^51^*.* The findings in this study demonstrate that cell-free systems are able to synthesize the individual subunits at equivalent or even higher concentrations (see Fig. 1 and 2). Nonetheless, our findings also indicated that the translocation efficiency emitted by the Mel signal peptide did not reach its full potential (see Fig. 2). As other studies have shown that Mel signal peptide is not always able to translocate every protein^52^, other signal peptides might be tested in the future. However, prior work has also shown that the Mel signal peptide is cleaved off of the synthesized protein, demonstrating the usability of the Mel signal peptide and eukaryotic cell-free systems^53^. The MS data acquired in this study depict that the A-subunit of two Shiga variants was successfully synthesized. A complete cleavage of the A-Subunits into A1 and A2 fragments could neither be clearly shown by autoradiography nor by mass spectrometry. This could be based on the methodological conditions of the SDS-PAGE and thus a potentially changed behavior of the samples within the gel. Future studies should synthesize the complete A-subunit as well as the A1 and A2 fragments individually and compare the different subunits. Such an experiment would further validate the processing capabilities within cell-free systems.

To our knowledge, up to now there are no reports on the cell-free synthesis of Stx proteins. Nonetheless, CFPS has already been used to assess the functionality of both purified Stx as well as bacterial cultures to identify Stx producing strains. In 1980, a rabbit reticulocyte as well as a wheat germ cell-free protein synthesis system was used to characterize the inhibitory effect of purified Shiga toxins. 50% inhibition was detected at 6 µg toxin/mL. This inhibitory effect could even be enhanced when the toxin was pretreated with urea and DTT or proteolytically treated^40^. In another study, an IC_50_ of 324 pg/µL Stx purified from *E. coli* strains was calculated to decrease luciferase activity in a rabbit reticulocyte cell-free system. Within the study, this methodology was further used to test culture supernatants to identify Stx-producing strains^41^. These studies already showed that an in vitro protein inhibition assay as applied in this study was useful to detect the functionality of Stx. Another study even identified differences in toxicity in different variants. A protein inhibition assay was performed by Skinner and colleagues in 2013 using rabbit reticulocyte lysates and showed that the Stx2f. variant was less toxic than the Stx2a variant as the calculated IC_50_s were 3.5 and 2.5 µg/mL, respectively^54^. In our study we used the CHO cell-free system to assess the effects of Stx variants as a mammalian system to further extrapolate upon the activity on human ribosomes and toxic effects on humans. The approach used here could therefore assess the overall total protein yield of the model protein, the qualitative reduction of protein using autoradiography as well as the functionality of the model protein Luc. Thus, our study could widen the analytical approach to assess the functionality of Stx. At 2 nM, which is equal to about 0.065 µg/mL, the StxA-subunit variants tested in this study significantly reduced the protein synthesis of Luc. This shows the efficiency of the used methods.

In this study in the in vitro protein inhibition assay solely the A-subunit was investigated. The gene sequences for Stx variants analyzed in this study derive from *S. dysenteriae* (Stx) and from *E. coli* (Stx2a). It can be postulated that the individual activity of the toxins differs due to structural differences in the variants. Crystal structures for the A-subunit of Stx and Stx2a have identified that the active domain in the A-subunit is blocked by the pentameric B-ring^55–57^. Thus, it will be necessary to assess the ribosome inactivating effect of the holotoxin as well. In prior studies it was shown that in the absence of the pentameric B-subunit, a conformational change of the A-subunit is triggered and the A2 domain is destabilized. This leads to the exposure of the active domain^58^. The data gathered in this study show that CFPS is a valid method for the screening of toxins and toxin variants in a time- and cost-efficient manner. This system can be transferred to diverse toxin groups and the analytical approach can be broadened to mutational analyses. As linear DNA templates can be used in cell-free systems^59^, DNA templates for truncated toxin fragments or templates incorporating site-directed mutations can be screened allowing the identification of active domains. Such studies have already been performed for cytotoxic proteins in cell-free systems^27,60^, and thus show the applicability of the system for future studies.

Toxic effects of Stx have already been investigated in prior studies. HeLa cell culture has been established as a model cell line for the assessment of Stx activity^36,61–63^. A study in 1980 showed a time and concentration dependent effect of Stx on HeLa cells. Primary inhibition of protein synthesis and DNA replication started 30 min after supplementing 50 ng toxin to HeLa cells while a secondary effect was seen for RNA synthesis^64^. A later study found that different Stx variants have a different effect on cells. Skinner and colleagues found that an overnight incubation of Stx2f. was less toxic on Vero cells than Stx2a. A cytotoxic dose of 50% was calculated at 1.6 pg/well and 0.3 pg/well for Stx2f. and Stx2a, respectively^54^. In order to validate the functionality of the holotoxins on viable cells an OPP-protein synthesis assay was performed in this study. Such assays are a quantitative alternative to asses protein synthesis rates within living cells and has been widely used to investigate the effects of bacterial toxins and biomedical research^65–67^. Our results demonstrated a significant reduction of protein synthesis in HeLa cells at concentrations of 10 nM (equal to ~ 715 pg/µL) and 5 nM (equal to ~ 357.5 pg/µL) holotoxin synthesized in *E. coli* and CHO-lysates, respectively. Both variants, Stx and Stx2a showed significant inhibition of protein synthesis. Thus, the concentrations used in our study were higher than in prior studies and future studies should assess whether purified toxins from cell-free synthesis reactions would enhance the toxic effect. Nonetheless, the in vitro protein inhibition assay clearly showed that a fast screening of the A-subunit at much lower concentrations was possible and therefore represent a fast and efficient way to assess the functionality of different Shiga toxins. As a prior study by Pellino has shown a preassembly of the Shiga AB_5_ complex is not necessarily needed to attack cells^50^. In our study the StxA-subunit synthesized in CHO lysate induced a significant reduction in protein synthesis in HeLa cells without the presence of the B-subunit which strengthens the findings of Pellino and others. Interestingly, another study has identified that a complete holotoxin of Stx1 and Stx2 was necessary to induce apoptosis in HeLa cells^63^. Our study showed that the holotoxin was active when both subunits were co-expressed but not when the subunits were individually expressed and mixed together subsequently. This phenomenon was already observed for other AB_5_ toxins which were synthesized in cell-free system, namely the cholera toxin and the heat-labile enterotoxin^27^. This might indicate that during the synthesis of the subunits together the close proximity to each other allows for a better assembly and nicking of the protein. As the single A-subunit was active in the in vitro protein translation inhibition assays, the effect of the toxin on cells might need other requirements that are not yet fully understood. Cell-free synthesized proteins might further be used to elucidate such pathways as the individual subunit might easily be modified by genetic mutations using PCR or the addition of fluorophores by click chemistry for intracellular tracking.

Taken together, this study demonstrates the ability to detect differences in toxicity among Stx variants using time-efficient methods. In vitro as well as cell-based approaches can be used to differentiate the toxic character of individual variants. The analysis of toxic variants from Shiga toxins will help to further understand the mechanisms of action of these toxins as well as will help to identify blockers for disease prevention.

## Material and methods

### DNA templates for cell-free protein synthesis

Gene sequences encoding Stx (derived from *Shigella dysenteriae*) with its catalytic A-subunit (Uniprot identifier: Q9FBI2) and its binding domain B (Uniprot identifier: Q7BQ98) were used modified for CFPS. Gene sequences encoding Stx2a of *E. coli* (UniProt identifier: P09385) were adapted for CFPS. Therefore, the sequences for the native signal peptides were removed. Sequences were designed according to Krebs et al. 2022^30^. Shortly, all sequences harbored a T7 promotor and T7 terminator. Templates for *E. coli*-based CFPS contained a Shine-Dalgarno sequence. Templates for CHO-based CFPS harbored an internal ribosomal entry site (IRES from the intergenic region of the cricket paralysis virus (CRPV)) to allow for a cap-independent translation initiation and the signal sequence from the honey bee toxin melittin (Mel) according to Thoring et al*.* 2017^32^. These sequences for both, *E. coli* and CHO-based synthesis, were obtained by de novo gene synthesis (Biocat GmbH) and cloned into the pUC57-1.8 K vector backbone. Plasmids were directly used for Stx in CHO-based cell-free synthesis. Plasmids for *E. coli*-based cell-free synthesis and CHO-based Stx2a synthesis were linearized the restriction enzyme BpmI (NEB) according to the manufacturer’s protocol and purified using DNA purification kit DNA Clean and Concentrator-25 (Zymo Research).

### Cell-free protein synthesis

Cell-free synthesis reactions were performed in two different lysates. Firstly, an *E. coli* cell lysate was prepared from *E. coli* BL21 Start (DE3, Invitrogen) as previously described^30^ and secondly, CFPS was performed using translationally active lysate derived from Chinese hamster ovary cells (CHO-K1, DSMZ) as previously described^31,32^. Protein synthesis was conducted in coupled transcription/translation reactions in a final volume of 20 to 80 μl. Templates for the Stx subunits were either synthesized individually to acquire the single subunit or the templates for the catalytic A-subunit and the binding subunit B were co-expressed in a 1:5 molar plasmid ratio. Further, an NTC was used as background control which only consisted of the translation mixture without the addition of any DNA template. Two different reaction formats were used within this study, namely a one-pot batch reaction for the synthesis characterization, functional characterization and antibody binding studies of the Stx as well as a CECF reaction for the visualization of the pentamer formation of the StxB-subunit. If required, reactions were supplemented with radioactively labeled ^14^C-leucine (Batch-based reactions: f.c. 50 μM, specific radioactivity 66.67 dpm/pmol, CECF-reactions: f.c. 50 μM, specific radioactivity 10 dpm/pmol, Perkin Elmer) to allow for qualitative and quantitative protein analysis (see below).

#### *E. coli*-based synthesis

Batch-based syntheses were performed according to Krebs et al*.* 2022^30^. Shortly, *E. coli* BL21 Star cells were grown in a shaking flask at 37 °C and 200 rpm in 2 × YPTG medium containing 10 g/L yeast extract, 16 g/L tryptone, 5 g/L NaCl, and 20 g/L glucose. T7 RNA polymerase was overexpressed and cells were harvested in the late log phase after a total of 4 h cultivation time. Cells were disrupted to generate a translationally active lysate. A mixture of 26% lysate was supplemented with HEPES buffer system, buffering ions, protease inhibitors, amino acids and an energy system. For the stabilization of linearized templates Streptavidin (Serva) was added to the reaction. Syntheses were incubated at 33 °C and 500 rpm for 2 h.

CECF reactions were conducted in a two chambered dialysis device (Biotech Rabbit) and incubated for 24 and 48 h at 33 °C and 600 rpm. The first chamber contained a reaction mixture similar to the batch-based reaction with the addition of sodium azide (f.c. 0.02%, Merck). Chamber two contained a feeding mixture that was composed of the HEPES–KOH buffer system and buffering ions, energy regenerating components, the caspase inhibitor and sodium azide.

#### CHO-based synthesis

Batch-based syntheses were performed according to standard protocols^47,68, 69^. Shortly, CHO-K1 cells (DSMZ) were cultivated in serum-free suspension cultivation in 5 L bioreactors (Sartorius Stedim Biotech GmbH)- using Power CHO-2 medium (Lonza). Cells were harvested in the exponential growth phase and disrupted for lysate preparation. Reactions composed of 40% (v/v) translationally active lysate were supplemented with a HEPES-KOH buffer system and buffering ions, energy regenerating components, T7 RNA polymerase, amino acids and PolyG for stabilization. Reactions were incubated in a thermomixer (Eppendorf) for 3 h at 30 °C and 500 rpm.

CECF reactions were conducted according to previously described protocols by Thoring et al. 2017 and Ramm et al. 2020^32,69^. Briefly, reactions were performed in a two chambered dialysis device (Biotech Rabbit) and incubated for 24 and 48 h at 30 °C and 600 rpm. Chamber one contained a reaction mixture similar to batch-based reactions with the addition of a caspase inhibitor AC-DEVD-CMK (30 µM; Promega) and sodium azide (f.c. 0.02%). Chamber two contained a feeding mixture that was composed of the HEPES–KOH buffer system and buffering ions, energy regenerating components, the caspase inhibitor and sodium azide.

### Protein fractionation

As the Stx subunits are soluble proteins, a fractionation of the crude translation mixture (TM) was performed to separate the protein from other components in the cell-free synthesis reaction. After the synthesis in the *E. coli* lysate, the TM was centrifuged (16,000×*g*, 10 min, 4 °C) resulting in the supernatant (SN) containing the soluble proteins and the pellet fraction (P) containing the accumulated protein.

As the StxA-subunit is a ribosome-inactivating protein, especially for eukaryotic ribosomes, the templates for the CHO-based cell-free synthesis harbored a Mel signal sequence. Hence, a translocation of the synthesized protein into the microsomal vesicles that are present in the CHO^32^ system was facilitated in order to allow for a protein synthesis within the eukaryotic system. Briefly, after the synthesis the TM was centrifuged (16,000×*g*, 10 min, 4 °C) resulting in the supernatant (SN) containing the soluble subunits that were not sufficiently translocated into the vesicles and the pelleted microsomes. The SN was removed and the pellet was resuspended in phosphate buffered saline (PBS, Merck) containing 0.5% of the mild detergent C3-((3-cholamidopropyl) dime-thylammonio)-1-propanesulfonate (CHAPS, Amresco) which was termed the microsomal fraction (MF). The MF was incubated at room temperature under rigorous agitation for 30 min and lastly centrifuged (16,000×*g*, 10 min, 4 °C) again. This step allowed the harvesting of soluble, translocated proteins present in the resulting second supernatant (SN2).

### Quantitative protein analysis

Total protein yields of cell-free synthesized proteins were determined by incorporation of ^14^C-leucine and subsequent precipitation by hot trichloro acetic acid (TCA, Carl Roth GmbH) as described previously^69^. Briefly, 3 µl aliquots of the cell-free synthesized toxin fraction were mixed with 3 ml of 10% TCA/ 2% casein hydrolysate (Carl Roth GmbH) solution and boiled at 80 °C for 15 min. Following a 30 min incubation on ice, non-incorporated ^14^C-leucine was removed using a vacuum filtration system (Hoefer) and the total protein yield of ^14^C-labeled proteins was determined by liquid scintillation counting (Hidex 600SL, Hidex). The total protein yield was calculated according to the following equations:

$$Protein\,yield \left[\frac{\mu g}{mL}\right]=\frac{scintillation\,counts\left[\frac{dpm}{mL}\right] * molecular\,weight\left[\frac{\mu g}{pmol}\right]}{specific\,radioactivity \left[\frac{dpm}{pmol}\right] * number\,of\,leucines}$$

$$\begin{aligned} & Specific \;radioactivity \left[ {\frac{dpm}{{pmol}}} \right] \\ & \quad = \frac{{stock\; concentration \;of {}_{ }^{14} C\; leucine*specific\; radioactivity\; of\; {}_{ }^{14} C\; leucine \;stock}}{Total \;concentration\; of\; leucine} \\ \end{aligned}$$

The total protein yield for co-expressed subunits was estimated using the sum of the molecular weight and the sum of the number of leucines of all expressed subunits. The quantitative analysis was performed in technical triplicates and error bars represent standard deviation.

### Qualitative protein analysis—autoradiography

Sodium dodecyl sulfate polyacrylamide gel electrophoresis (SDS-PAGE) using precast gels (NuPAGE, 10% Bis-Tris, Life technologies) was performed to confirm the molecular mass of radio-labeled cell-free synthesized toxins. Therefore, 3 µl aliquots of the toxin fraction were precipitated in cold acetone (Carl Roth GmbH) as described previously^69^. Protein samples were dissolved in LDS samples buffer (NuPAGE, Life technologies) and SDS-PAGE was performed under non-reducing and reducing (addition of 50 mM DTT, Aplichem GmbH) conditions. SeeBlue™ Plus2 protein standard and Page Ruler Unstained (Thermo Fisher Scientific Inc.) were used as a protein size determination. Proteins were separated on precast NuPAGE SDS-PAGE gels for 35 min at 185 V and were stained with a self-prepared Coomassie Brilliant Blue G250 solution (Serva, 75 mg/L and 35 mM HCL, Carl Roth GmbH). Subsequently, gels were dried on Whatman paper for 70 min at 70 °C (Unigeldryer 3545D, Uniequip) and dried gels were exposed to phosphor screens (GE Healthcare) for a minimum of three days. The protein standard bands were marked using ^14^C-Leucin ink to be detectable on the autoradiographs. Finally, radio-labeled proteins were visualized using a phosphor imager system (Amersham RGB Imager, GE Healthcare).

### Qualitative protein analysis—western blotting

To detect the Stx subunits, a Western Blot analysis was performed. SDS-Gels were prepared as described above and blotted using an iBlot 2 Gel Transfer Device (Life Technologies) and Nitrocellulose iBlot Gel Transfer Stacks (Invitrogen). Blotting was performed for 10 min. 0.1% Tris Buffered Saline (TBS, composed of Tris (PanReac) and NaCl (PanReac) and puffered to a pH of 7.5 with HCl)-Tween solution (TBS-T, Tween-20 Applichem) was used for washing membranes three times. Subsequently, membranes were blocked using 2% (w/v) BSA (Sigma Aldrich) TBS-T solution over night at 4 °C. A primary mouse monoclonal antibody was tested, namely the Shiga toxin 2 Antibody 11E10 (sc-52727, Santa Cruz). The antibody was diluted 1:1000 in 1% (w/v) BSA TBS-T solution and the membranes were incubated for 2 h at room temperature (RT). After washing the membranes three times with TBS-T, the secondary antibody Horse Radish Peroxidase (HRP)-conjugated goat anti-mouse IgG (Azure Biosystems) 1:3000 in 1% (w/v) BSA TBS-T solution. Membranes were incubated with the secondary antibody for 1.5 h at RT. The HRP coupled to the secondary antibody, was used for detection by WesternBright ECL HRP (Biozym). Illumination signal was detected using a AzureWestern C600 Imaging System (Azure Biosystems).

### Cell-based StxA2a-His expression

Recombinant StxA2a-His was expressed using *E. coli* C43 (DE3) pET22b( +)/StxA2a-His in autoinduction medium ZYM-5052 as described earlier^36^. The expression culture was inoculated with a 1:50 dilution of an overnight culture of the expression strain grown in Luria–Bertani (LB)-broth, containing a final concentration of 100 µg/mL ampicillin, 37 °C, 180 rpm. The expression culture was incubated at 37 °C with 180 rpm until an optical density at 600 nm (OD_600_) of 1.3 to 1.5 was obtained. The temperature was then decreased to 20 °C and the culture was further incubated for 20 h with 180 rpm. Cells were harvested by centrifugation at 5,000 × g for 10 min at 4 °C (Avanti J25 centrifuge, Beckman Coulter). The pellet was washed with PBS (8 g/L NaCl, 0.2 g/L KCl, 1.78 g/L Na_2_HPO_4_, 0.24 g/L KH_2_PO_4_), resuspended in His-binding buffer (6.1 g/L Tris-base, 17.35 g/L NaCl)^36^, supplemented with Pierce™ EDTA-free proteinase inhibitor (Thermo Scientific) and lysozyme (1 mg/mL) and incubated on ice for 45 min. Cells were lysed by sonication (6 cycles with 10 son, 20 s off, 70% amplitude, Sonifier®, with a microtip), and the soluble protein was separated from the cell debris by centrifugation at 25,000×*g* for 45 min, 4 °C. The expressed StxA2a-His was purified first by Immobilized Metal Affinity Chromatography. The supernatant was applied to a PD-10 column (GE Healthcare) with 1 mL Ni^2+^-NTA agarose (Qiagen) as stationary phase and incubated for 16 h at 4 °C. The column was washed three times with 2 mL His-binding buffer with increasing imidazole concentrations (10 mM, 20 mM, 40 mM) and StxA2a-His was eluted three times with His-elution buffer containing 250 mM imidazole (2 mL, 1 mL and 0.5 mL, see Supplementary Fig. 11a). StxA2a-His was further purified by Size Exclusion Chromatography using a HighLoad™ 16/600 Superdex™ 75 pg column (180 mL, GE Healthcare, see Supplementary Fig. 11b). The fractions containing the purified StxA2a-His were collected and concentrated to a final volume of 1 mL using an Amicon ultra centrifugal filter. Purification was confirmed by SDS gel electrophoresis as previously described for recombinant subtilase subunits^70^.

### Determination of protein concentration

The protein concentration of pure StxA2a-His was measured spectrophotometrically using a NanoDrop 2000 device (Thermo Scientific). The absorption was measured at 280 nm and the protein concentration was calculated with the theoretical extinction coefficient (33.140 M^−1^ cm^−1^) and the molecular mass (35.7 kDa) of StxA2a-His calculated by ProtParam (https://web.expasy.org/protparam/).

### In vitro protein translation inhibition assay

As a first step to assess the functionality of the StxA-subunit, a so-called protein translation inhibition assay was performed using the cell-free system. The StxA- and StxA2a-subunits were synthesized in a cell-free manner (either using an *E. coli* or CHO lysate) as described above. Two identically treated reactions were parallelly pipetted with the only difference that one contained ^14^C-labled leucine and the second reaction did not. After the synthesis the reactions were fractionated as described (*E. coli*-based synthesis—SN1 fraction; CHO-based synthesis—SN2 fraction). ^14^C-labeled samples were used for quantification of total protein yield. Subsequently, the non-labeled StxA samples at a defined concentration range of 0.002 to 2 nM were added to the cell-free synthesis of the model protein Luciferase (Luc). The Luc synthesis was also performed in both cell-free systems. As control reactions of the Luc synthesis in the presence of cell-free synthesized StxA the following reactions were used: additional Luc syntheses supplemented with equivalent volumes of PBS, PBS/0.5% CHAPS, NTC fractions, ddH_2_O as well as a cell-based StxA1 control protein at a concentration range of 0.002 to 200 nM. 

In the end, the total protein yield of de novo synthesized Luc, the Luc activity as well as the qualitative Luc protein band in the autoradiograph were assessed.

### Luc activity assay

Luminescence of Luc was measured using LAR reagent (Promega). The substrate contained in this mixture is converted by luciferase emitting luminescence signal in this process. 50 µL of LAR reagent was added to 5 µL of the TM of Luc reaction in a white 96-well plate (Thermo Scientific). The overall luminescence was measured using a FLUOstar OMEGA plate reader (BMG Labtech). Optimal relative light unit (RLU) values were achieved using 1,600 gain settings. Measurements were performed in two biological replicates with two technical replicates each (n = 4).

### Cell-based toxicity studies

For the cell-based protein synthesis assay HeLa cells (1*10^5^ cells/well) were seeded into 24-well microtiter plates and incubated for 24 h (37 °C; 5% CO_2_). Cells were intoxicated with 10 nM of Stx variants produced in *E. coli* lysates or 5 nM of Stx variants produced in CHO lysates for 18 h (37 °C; 5% CO_2_). Therefore, the individual Stx- and Stx2a-subunits (StxA and StxB) as well as the co-expressed subunits (holotoxin) were synthesized in a cell-free manner (either using an *E. coli* or CHO lysate) as described above. Two identically treated reactions were parallelly pipetted for each sample while one contained ^14^C-labled leucine and the second reaction did not. After the synthesis the reactions were fractionated as described (*E. coli*-based synthesis—SN1 fraction; CHO-based synthesis—SN2 fraction). ^14^C-labeled samples were used for quantification of total protein yield while non-labelled protein samples were supplemented to the cell-based analysis. In addition, since diphtheria toxin (DT) is a known inhibitor of protein synthesis, cells were intoxicated with 1 nM DT to serve as a positive control for the assay. Untreated cells (Ctrl) served as control for maximal OPP-incorporation. Cells without OPP served as negative control for background fluorescence. As additional control, cells were incubated with the NTC fractions. Changes in protein synthesis were determined by using the Click-&-Go™ Plus OPP Protein Synthesis Assay Kit (Click Chemistry Tools) following the manufacturer´s protocol. Briefly, after intoxication, the cells were labelled with 5 µM OPP for 30 min, collected by scratching in OPP-containing medium and transferred into 1.5 mL reaction tubes. The cells were centrifuged at 500 rcf for 3 min and the supernatant was discarded. The obtained cell pellet was washed with 1% BSA in PBS at 500 rcf for 3 min (wash step), followed by fixation in 4% paraformaldehyde (Merck) at RT. This was followed by an additional washing step and permeabilization of the cells in a saponin-containing permeabilization buffer (0.1% saponin, 0.5% BSA, 0.01 NaN_3_) for 15 min at RT. Subsequently, the cells were centrifuged (500 rcf; 3 min) and stained in 100 µL of the reaction cocktail (prepared with reagents and as recommended by the manufacturer; the cocktail includes the green fluorescent AZDye 488 Azide Plus) for 20 min in the dark at RT. After two additional washing steps, the cells were resuspended in PBS and green fluorescence of cells was measured as direct measure for OPP-incorporation and protein synthesis using BD FACSCelesta™ device (Blue laser 488 nm; 530/30 filter). Flow cytometric data was visualized and analyzed by using the open source Flowing Software (Turku Bioscience Centre). Cells were plotted in an FSC-A/FSC-H dot plot for excluding cell duplicates. A green fluorescence histogram was used to discriminate between OPP-incorporating and -non-incorporating cells and for quantification of the respective cell populations.

### Statistical analysis

A univariate analysis of variance was used to assess the significant changes in the in vitro translation inhibition assays for Stx proteins regarding the total protein yield of the Luc and the Luc activity. The Origin 2021 software was used to perform the ANOVA. Significance was tested according to both Bonferroni and Tuckey. Three thresholds for the probability of an error (*p*-value) were used to define statistically significant changes. A significantly different change (*) was defined at *p* < 0.05, a very significantly different change (**) at *p* < 0.01 and a highly significantly different change (***) at *p* < 0.001.

### Mass spectrometry analysis

StxA and StxA2a were synthesized in CHO- and *E. coli* based lysates as described above. Samples were loaded onto 10% Bis-Tris SDS-PAGEs. Proteins were cut from the gel as depicted in Supplementary Fig. 12 and send for mass spectrometry analysis at the Core Facility Hohenheim (University of Hohenheim, Stuttgart, Germany).

Proteins were in-gel-digested using trypsin (Roche) according to Shevchenko et al.^71^. After digestion, the supernatant was collected in a new Eppendorf tube, dried down in a vacuum centrifuge and stored at − 20 °C. Dried samples were dissolved in 0.1% TFA for nano-LC–MS/MS analysis.

Nano-LC–ESI–MS/MS experiments were performed on an Ultimate 3000 nano-RSLC (Thermo Fisher Scientific) coupled to an Exploris 480 mass spectrometer (Thermo Fisher Scientific) using a Nanospray-Flex ion source (Thermo Fisher Scientific). Tryptic peptides were directly injected to a pre-column (µ-pre-column C18 PepMap100, 300 µm, 100 Å, 5 µm × 5 mm, Thermo Fisher Scientific) and then separated on a NanoEase analytical column (NanoEase M/Z HSS C18 T3, 1,8 µm 100 Å 75 µm × 250 mm column, Waters GmbH) operated at constant temperature of 35 °C. Gradient elution was performed at a flow rate of 300 nl/min using a 30 min gradient with the following profile: 2%–5% solvent B in 4 min, 5%–27% solvent B in 30 min, 27%–45% solvent B in 5, 45%–96% solvent B in 3 min, isocratic at 96% solvent B for 10 min, 95%–5% solvent B in 8 min and 5%–2% solvent B in 2 min followed by 3 min column re-equilibration at 2% solvent B. Solvents used were 0.1% formic acid (solvent A) and 0.1% formic acid in acetonitrile/H_2_O (80/20, v/v, solvent B).

The Exploris 480 and the Ultimate 3000 nano-RSLC were operated under the control of Xcalibur 4.4.16.14 and SII Xcalibur 1.6.0.6893 software. MS spectra (m/z = 300–1500) were detected in the Orbitrap at a resolution of 60.000 (m/z = 200) using a maximum injection time (MIT) of 50 ms and an automatic gain control (AGC) value of 3 × 10E6. Internal calibration of the Orbitrap analyzer was performed using lock-mass ions from ambient air as described in Olsen et al.^72^. Data dependent MS/MS mass spectra were generated for the 10 most abundant peptide precursors in the Orbitrap using high energy collision dissociation (HCD) fragmentation at a resolution of 15.000 (m/z = 200) and a normalized collision energy of 30. Further settings for MS/MS spectra included an isolation width of 1.6 Da, a MIT of 50 ms and an automatic gain control (AGC) value of 5 × 10E4.

### MS data analysis

Mascot 2.6 (Matrix Science) was used as search engine for protein identification. Spectra were searched against the *Escherichia coli* K12 (UP000000625) and Cricetulus griseus (UP000001075) reference proteome sequences downloaded in FASTA-format from UniProt. Sequences of Shiga toxin subunit A (StxA, Uniprot accession number: Q9FBI2) and Shiga-like toxin 2 subunit A (Stxa 2a, P09385) were added to the reference sequences^73^. Search parameters specified trypsin as cleaving enzyme, a 5 ppm mass tolerance for peptide precursors and 0.02 Da for fragment ions. Carbamidomethylation of cysteine residues was defined as fixed modification and Methionine oxidation was allowed as variable modification.

Mascot search results were imported into Scaffold version 4.10. (Proteome Software). Peptide identifications were accepted with a peptide probability greater than 95.0% as specified by the Peptide Prophet algorithm^74^. Proteins had to be identified by at least two peptides and a protein probability of at least 99.0% to be accepted. Protein probabilities were assigned by the Protein Prophet algorithm^75^.

## Supplementary Information

Supplementary Information.

## Data Availability

All relevant data are within the paper and its Supporting Information files.
