# Supplementary material for: Synthesis of biologically active Shiga toxins in cell-free systems
Source: Sci Rep. 2024 Mar 13;14:6043. doi: 10.1038/s41598-024-56190-3 (PMC11636806; doi:10.1038/s41598-024-56190-3)
Supplement: Supplementary file 1 — Supplementary Information. [file 41598_2024_56190_MOESM1_ESM.docx]

**Supplementary Information**

**Synthesis of biologically active Shiga toxins in cell-free systems**

Franziska Ramm^1*#^, Danny Kaser^1,2#^, Irina König^3^, Juliane Fellendorf^4^, Dana Wenzel^1^, Anne Zemella^1^, Panagiotis Papatheodorou^3^, Holger Barth^3^, and Herbert Schmidt^4^*

#Franziska Ramm and Danny Kaser contributed equally to this work.

*Franziska Ramm and Herbert Schmidt share the corresponding authorship

*Affiliations*

^1^Fraunhofer Institute for Cell Therapy and Immunology (IZI), Branch Bioanalytics and Bioprocesses (IZI-BB), Am Mühlenberg 13, 14476 Potsdam, Germany.

^2^University of Potsdam, Institute of Nutritional Science – Nutritional Toxicology, Arthur-Scheunert-Allee 114-116, 14558 Nuthetal, Germany

^3^Institute of Experimental and Clinical Pharmacology, Toxicology and Pharmacology of Natural Products, Ulm University Medical Center, Albert-Einstein-Allee 11, 89081 Ulm, Germany.

^4^Department of Food Microbiology and Hygiene, Institute of Food Science and Biotechnology, University of Hohenheim, Garbenstraße 28, 70599 Stuttgart, Germany.

To whom correspondence should be addressed:

Franziska Ramm: [Franziska.ramm@izi-bb.fraunhofer.de](mailto:Franziska.ramm@izi-bb.fraunhofer.de)

Herbert Schmidt: [herbert.schmidt@uni-hohenheim.de](mailto:herbert.schmidt@uni-hohenheim.de)

**Supplementary Information**

**
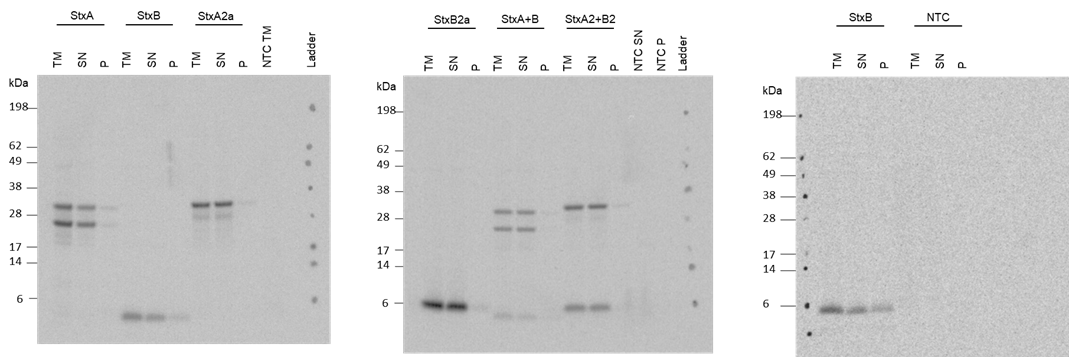
**

**Supplementary Figure 1: Uncropped autoradiographs from *E. coli*-based cell-free Stx synthesis.** Stx subunits variants were synthesized in a batch reaction in *E. coli* lysate. Qualitative analysis by autoradiography showing ^14^C‐leucine labeled Stx proteins.

**
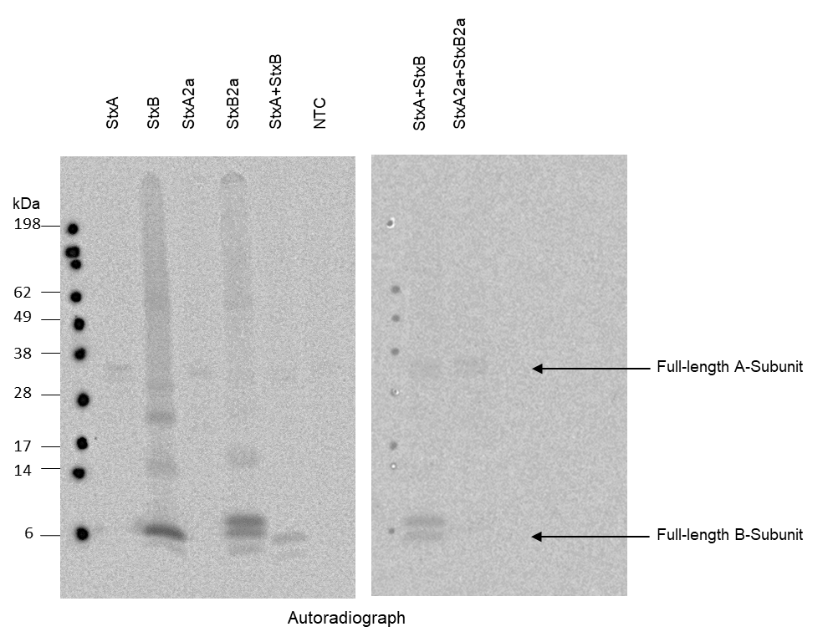
**

**Supplementary Figure 2: Uncropped autoradiographs from CHO-based cell-free Stx synthesis.** Stx subunits variants were synthesized in a batch reaction in *E. coli* lysate. Qualitative analysis by autoradiography showing ^14^C‐leucine labeled Stx proteins.

**
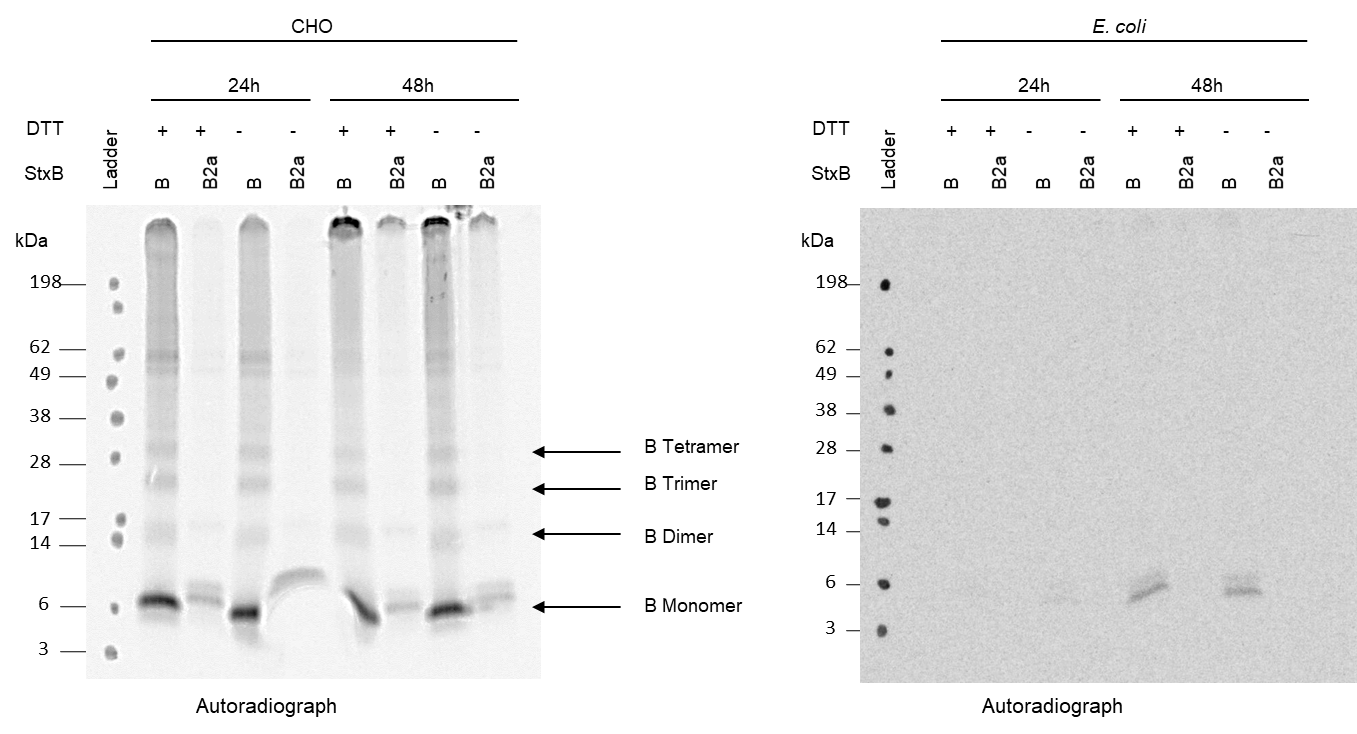
**

**Supplementary Figure 3: Multimerization of StxB in cell-free systems.** StxB variants were synthesized in a CECF reaction either in CHO or *E. coli* lysate for 24 or 48h. The multimerization was investigated under non-reducing and reducing (addition of 50 mM DTT) conditions. Qualitative analysis by autoradiography showing ^14^C‐leucine labeled StxB proteins.


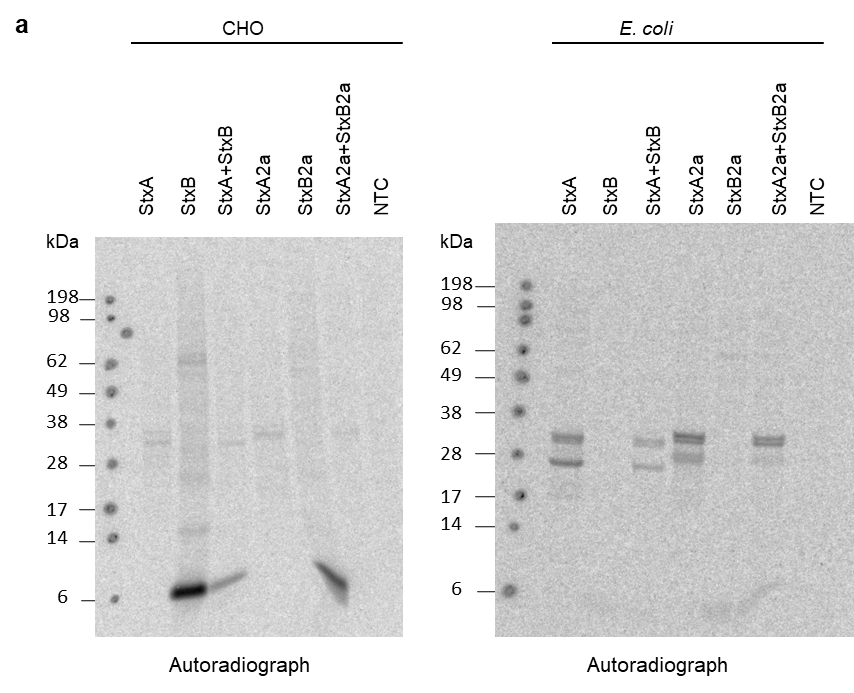


**Supplementary Figure 4: Western blot detection of Stx subunits.** Stx single subunits were synthesized in CHO (left) or *E. coli* (right) lysate either separately or in a co-expression (1:5 molar plasmid ratio, StxA+StxB and StxA2a+StxB2a). a) Qualitative analysis by autoradiography showing ^14^C‐leucine labeled on western blot of Stx2 antibody.


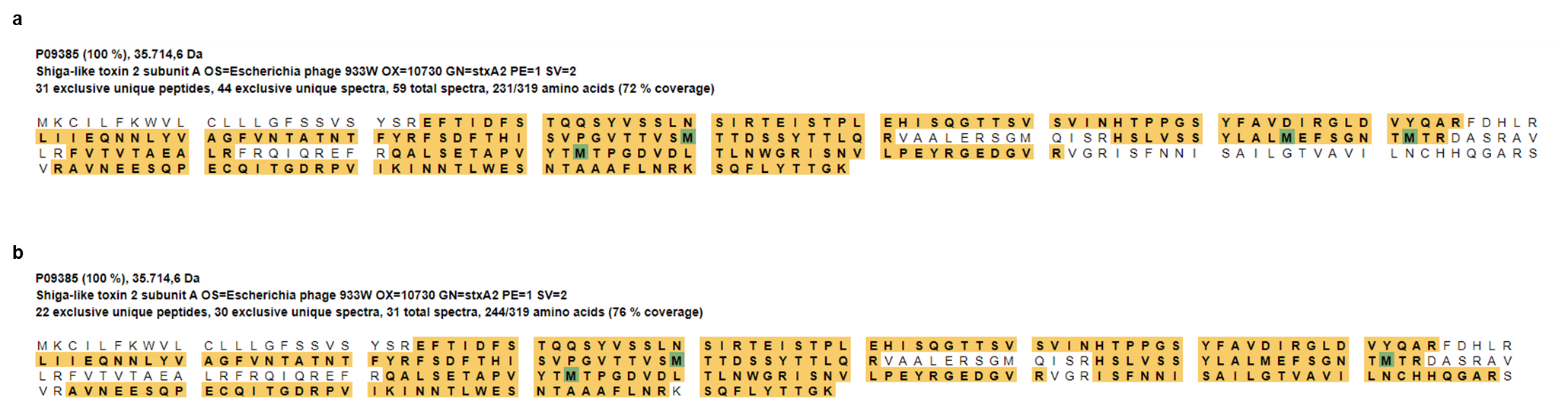


**Supplementary Figure 5: Peptide analysis StxA2a synthesized in *E. coli* lysate.** Coverage of peptides identified by mass spectrometry of a) StxA2a complete protein and b) potentially cleaved StxA2a.


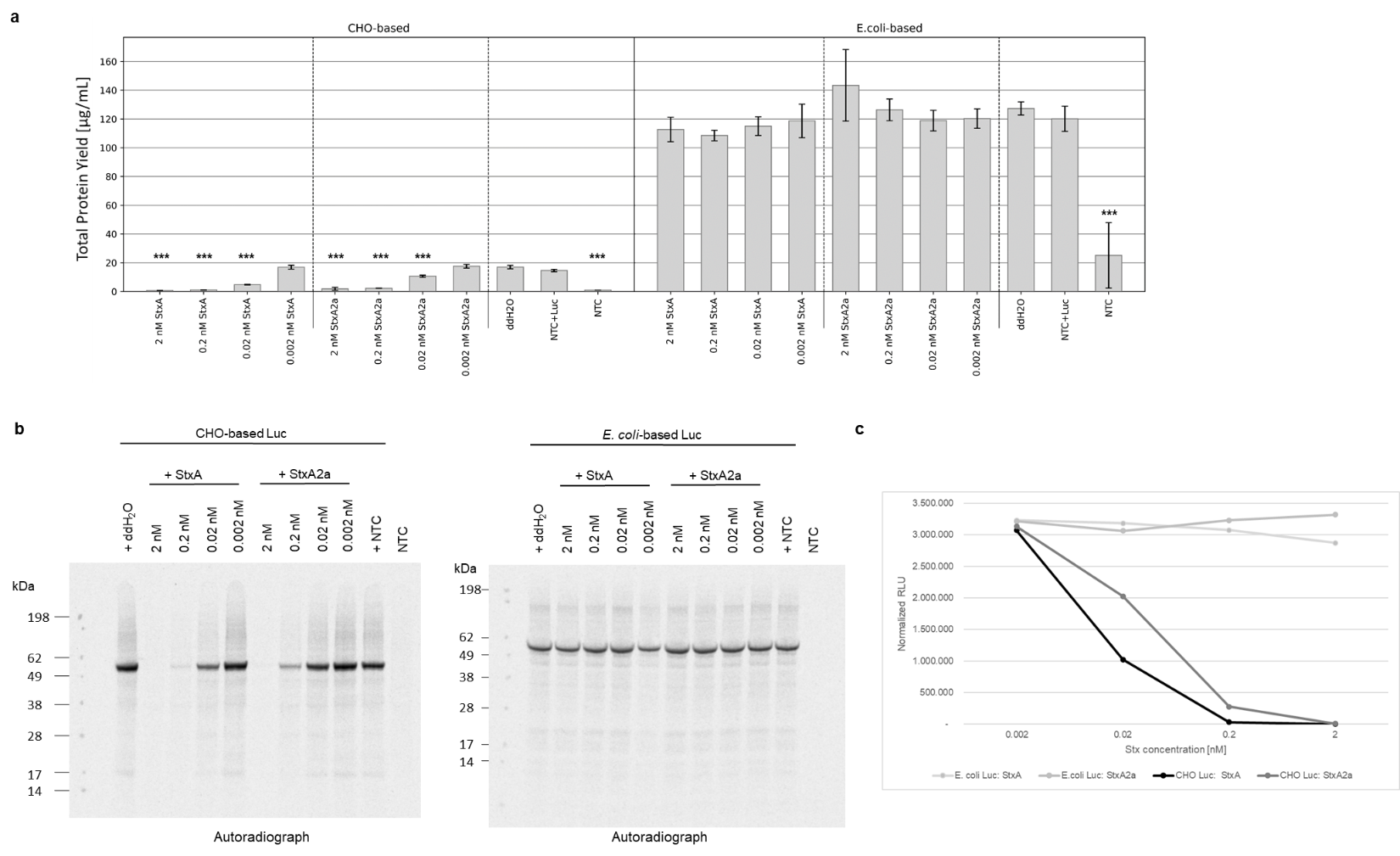


**Supplementary Figure 6: Establishing the in vitro protein inhibition assay.** Luc was synthesized in an *E. coli*- and CHO based cell-free system. The addition of water or NTC was monitored as negative controls and StxA variants (synthesized in *E. coli* lysate) at a concentration range of 0.002 to 2 nM were added to assess the protein synthesis inhibition ability. a) Quantitative analysis of synthesized Luc by liquid scintillation counting. Standard deviations were calculated from triplicate analysis. Statistical significance by ANOVA according to Bonferroni and Tuckey as indicated by * as compared to Luc + ddH_2_O. b) Qualitative analysis by autoradiography showing ^14^C‐leucine labeled Luc. c) Luc activity as measured by relative light units (RLU). RLU were normalized to the volume used in the assay as 5 µl were used in after the CHO-based Luc synthesis and 2.5 µl were used after the *E. coli*-based Luc synthesis.


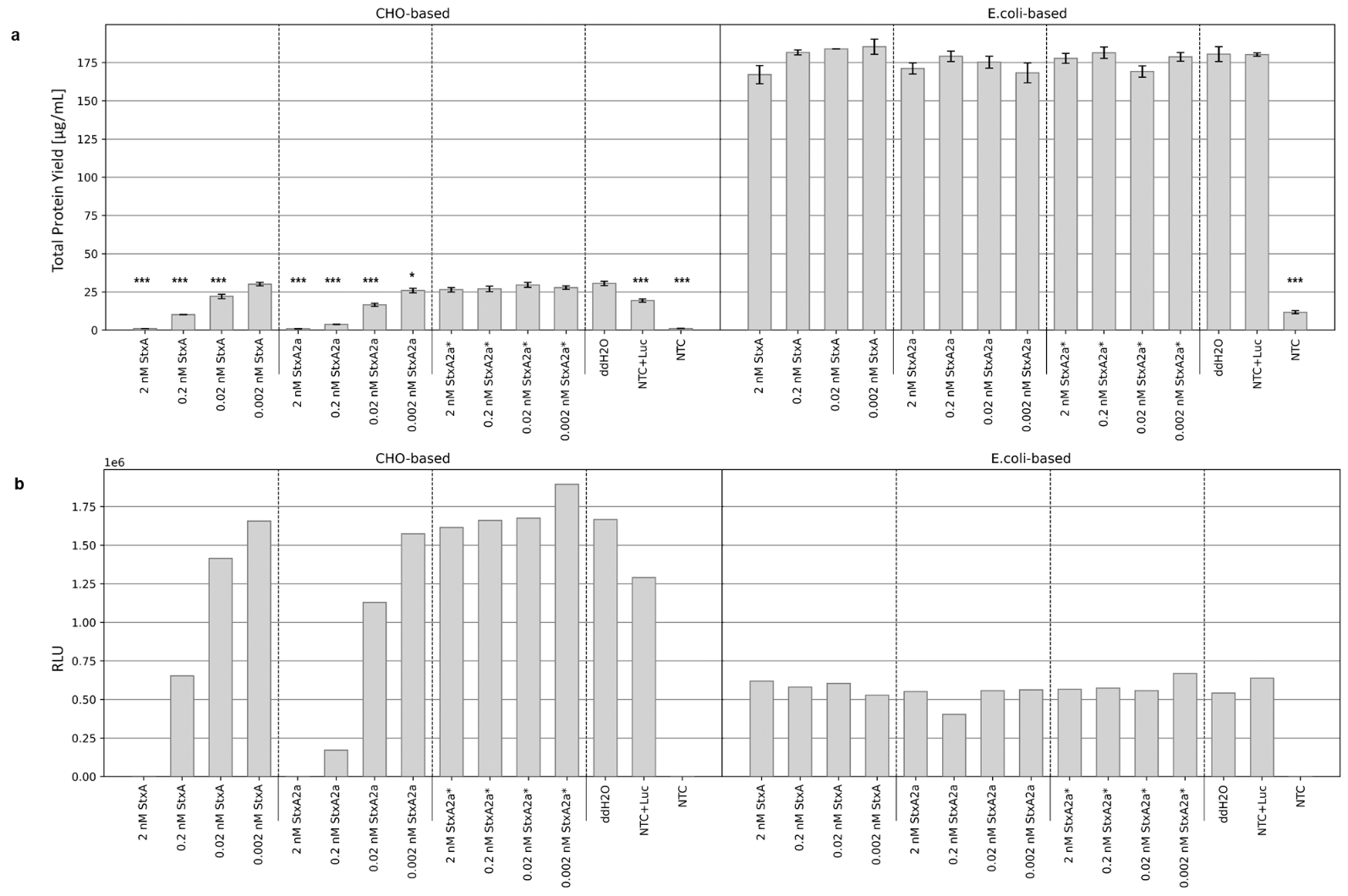


**Supplementary Figure 7: *In vitro* protein inhibition with Stx variants from an *E. coli* cell-free system**. Luc was synthesized in an *E. coli*-and CHO-based cell-free system. The addition of water or NTC was monitored as negative controls and StxA variants (StxA and StxA2a) at a concentration range of 0.002 to 2 nM as well as the positive control, StxA2a from cell-based *E. coli* synthesis (StxA2a*) were added to assess the protein synthesis inhibition ability. a) Quantitative analysis of synthesized Luc by liquid scintillation counting. Standard deviation derived from two assays with triplicate analysis (n = 6). Statistical significance by ANOVA according to Bonferroni and Tuckey as indicated by * as compared to Luc + ddH_2_O. b) Luc activity as measured by relative light units (RLU).


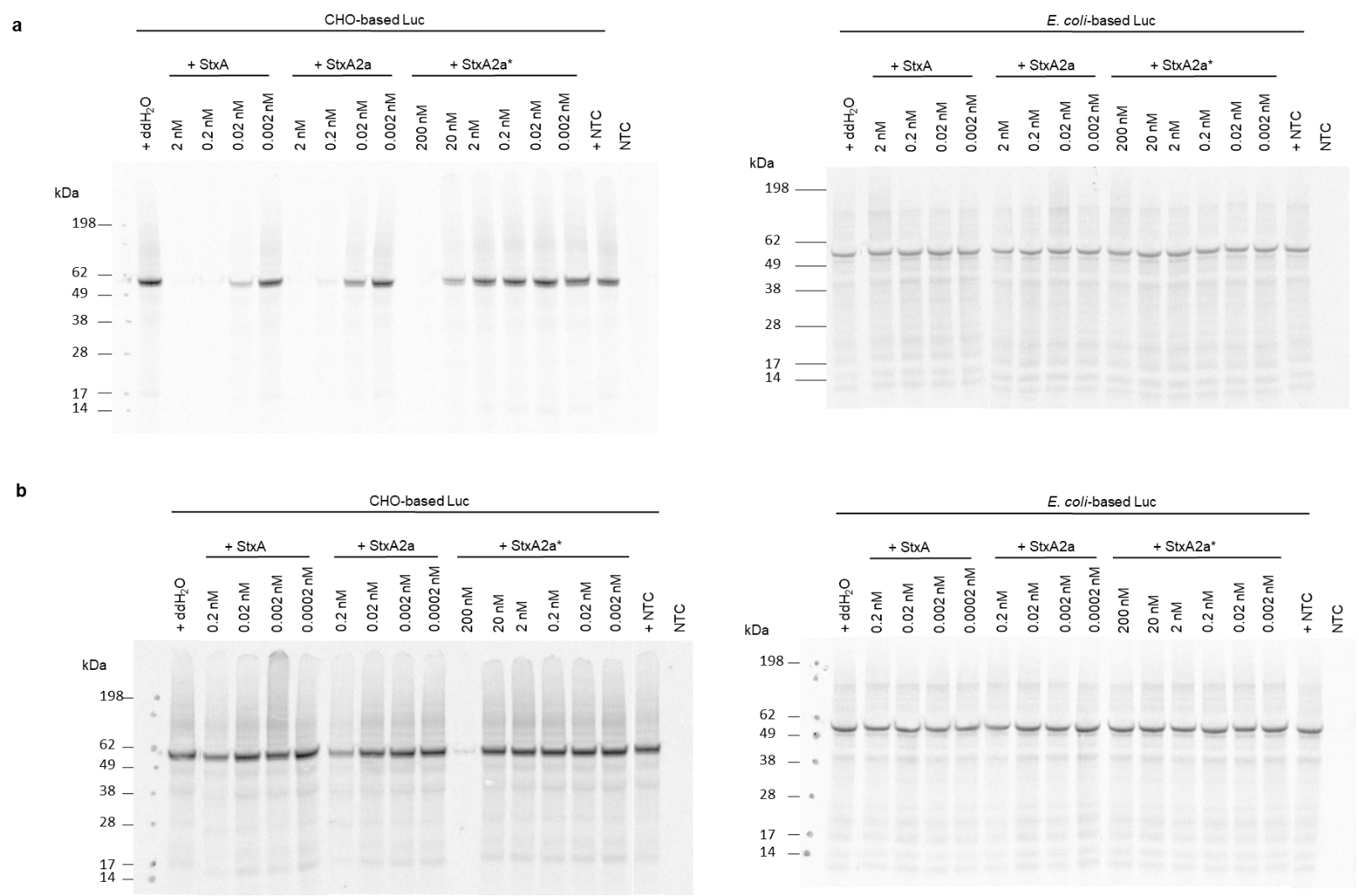


**Supplementary Figure 8: Complete autoradiographs from Figures 4 and 5.** Luc was synthesized in an *E. coli*- or CHO-based cell-free system. The addition of water or NTC was monitored as negative controls and StxA variants (StxA and StxA2a) at a concentration range of 0.0002 to 2 nM as well as the positive control, StxA2a from cell-based *E. coli* synthesis (StxA2a*) were added to assess the protein synthesis inhibition ability. Qualitative analysis via autoradiography of Luc protein samples after the addition of StxA samples after *E.coli*- (a) or CHO (b)-based cell-free synthesis.


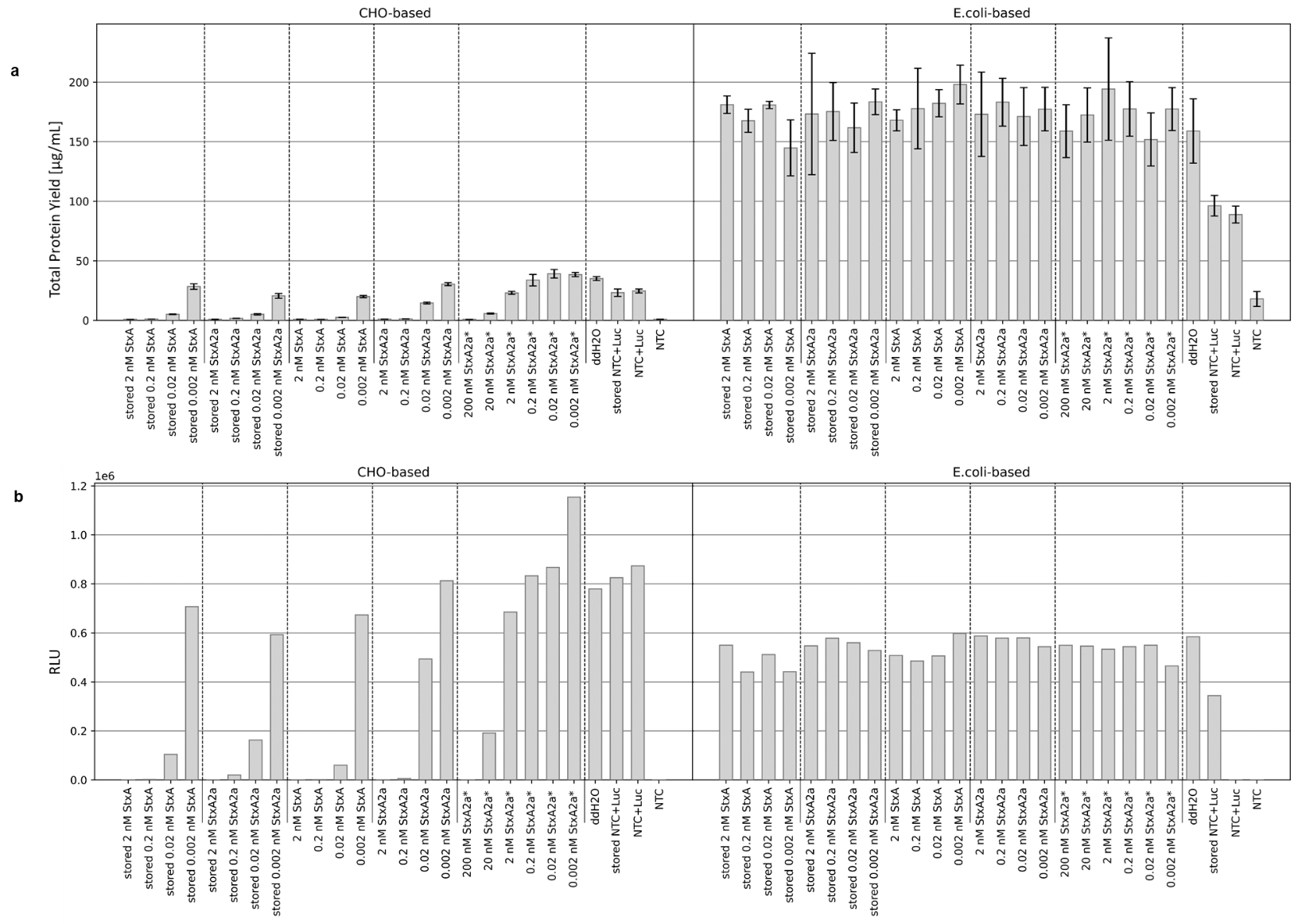


**Supplementary Figure 9: Storing experiment for the in vitro protein inhibition assay of Stx synthesized in *E. coli* lysate.** Luc was synthesized in an *E. coli*- and CHO-based cell-free system. The addition of water or NTC was monitored as negative controls and StxA variants at a concentration range of 0.002 to 2 nM were added to assess the protein synthesis inhibition ability. The positive control, StxA2a from cell-based *E. coli* synthesis (StxA2a*) was tested in a range of 0.002 to 200 nM. Stored StxA and StxA2a were synthesized the same day as the cell-based E. coli samples (StxA2a*) and stored over the same time. Cell-free synthesized StxA and StxA2a were freshly prepared before the assay. a) Quantitative analysis of synthesized Luc by liquid scintillation counting. Standard deviations were calculated from triplicate analysis. b) Qualitative analysis by autoradiography showing ^14^C‐leucine labeled Luc. c) Luc activity as measured by relative light units (RLU).


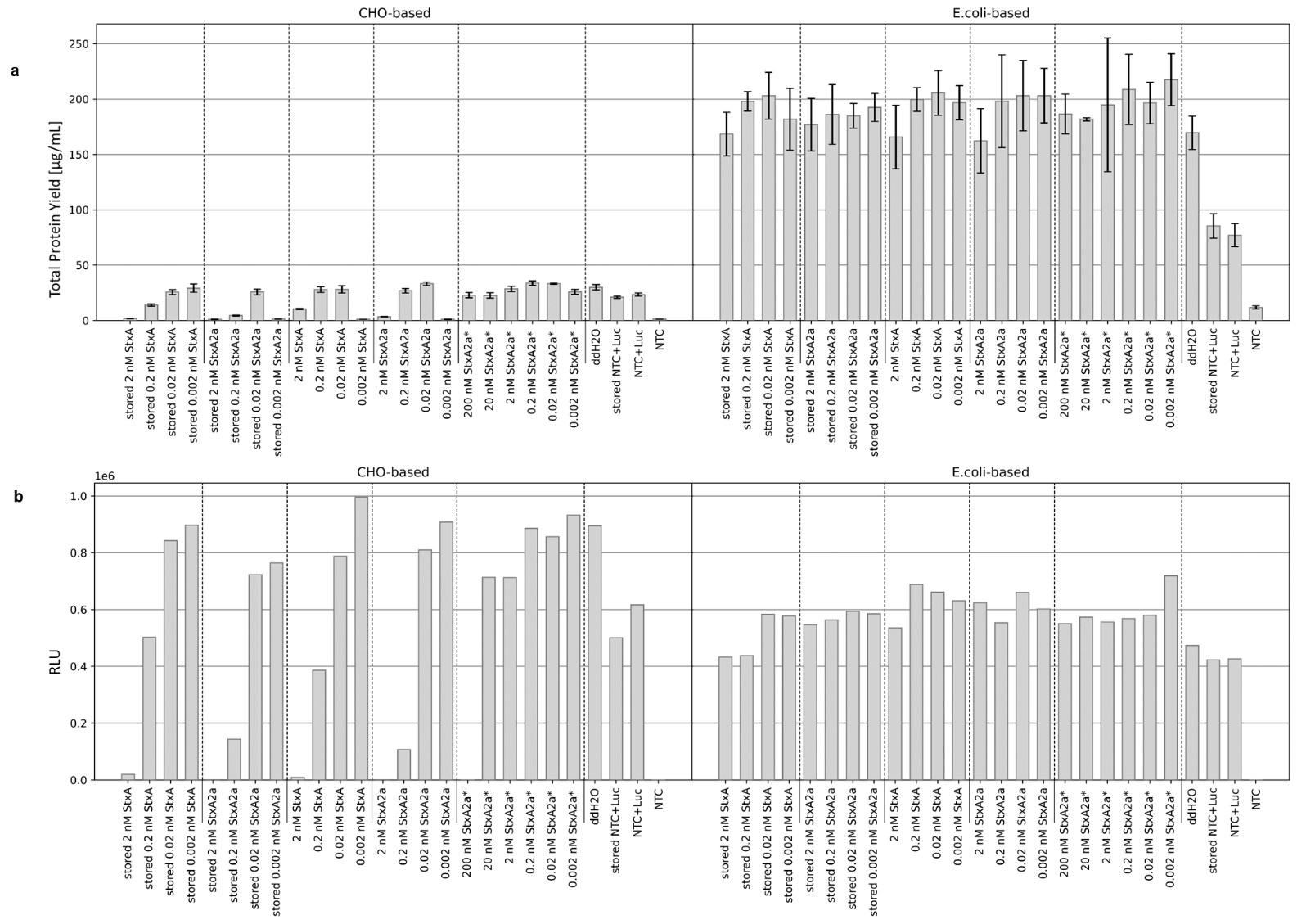


**Supplementary Figure 10: Storing experiment for the *in vitro* protein inhibition assay of Stx synthesized in CHO lysate.** Luc was synthesized in an *E. coli*- and CHO-based cell-free system. The addition of water or NTC was monitored as negative controls and StxA variants at a concentration range of 0.002 to 2 nM were added to assess the protein synthesis inhibition ability. The positive control, StxA2a from cell-based *E. coli* synthesis (StxA2a*) was tested in a range of 0.002 to 200 nM. Stored StxA and StxA2a were synthesized the same day as the cell-based E. coli samples (StxA2a*) and stored over the same time. Cell-free synthesized StxA and StxA2a were freshly prepared before the assay. a) Quantitative analysis of synthesized Luc by liquid scintillation counting. Standard deviations were calculated from triplicate analysis. b) Qualitative analysis by autoradiography showing ^14^C‐leucine labeled Luc. c) Luc activity as measured by relative light units (RLU).


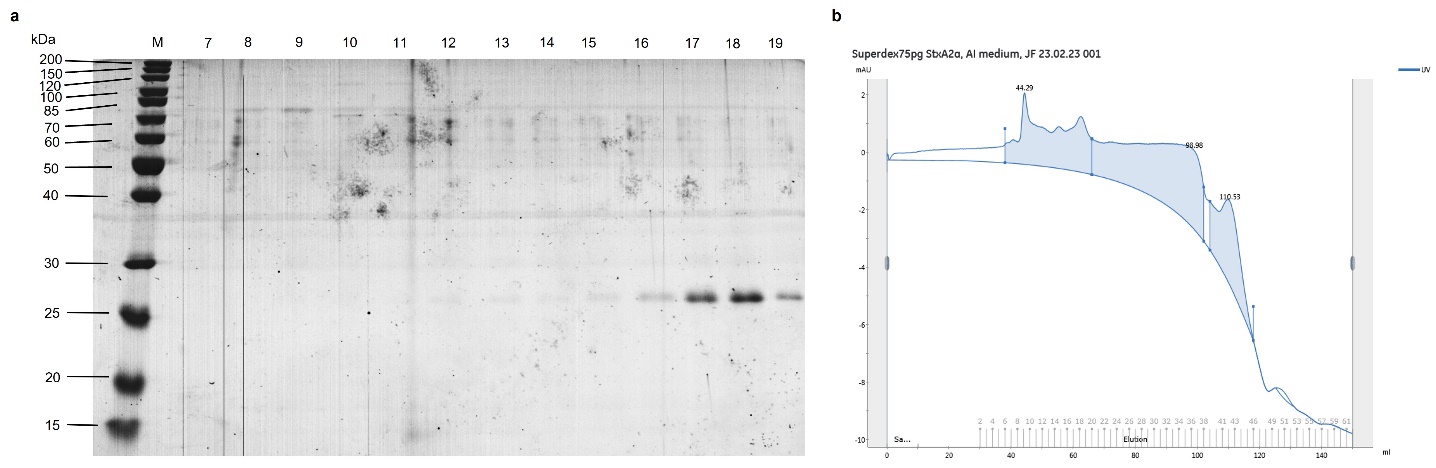


**Supplementary Figure 11: Cell-based StxA2a expression.** Recombinant StxA2a-His was expressed using *E. coli* C43 (DE3) pET22b(+)/StxA2a-His in autoinduction medium ZYM-5052. a) SDS-PAGE from the purified StxA2a-His after His-tag purification. Number indicate elution fractions. b) Chromatogram of StxA2a sample after Size Exclusion chromatography using a HighLoad^TM^ 16/600 Superdex^TM^ 75pg column.


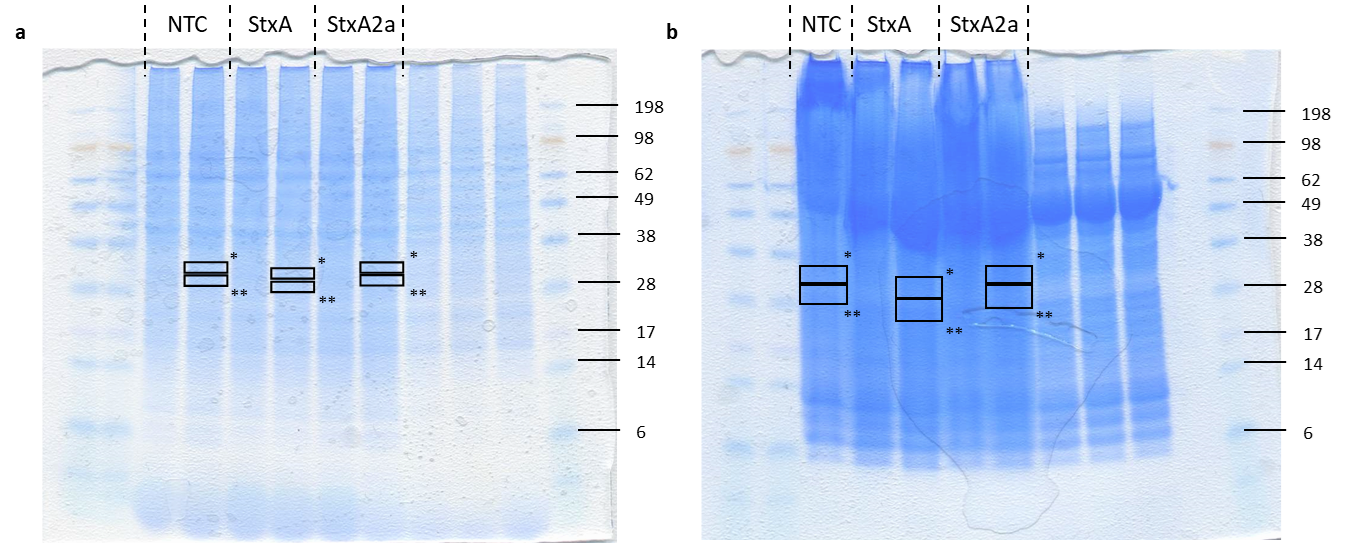


**Supplementary Figure 12: Original SDS-PAGE for sample preparation for mass spectrometry analysis for CHO- (a) and *E. coli*-based (b) samples.** Excised gel slices are marked with black boxes. * - Region corresponding to complete StxA or StxA2a; ** - Region corresponding to potentially cleaved StxA or StxA2a subunit.

**Supplementary Table 1**: Identification parameters of mass spectrometry analysis from Scaffold-Analysis for StxA and StxA2a from CHO-based cell-free synthesis.

| Protein | Sample | Accession | Coverage | Probability | Percentage of total Spectra | Exclusive unique peptides | Exclusive unique spectra count | Total spectra count | MW |
| --- | --- | --- | --- | --- | --- | --- | --- | --- | --- |
| Shiga toxin subunit A | StxA | Q9FBI2 | 44.90% | 100 % | 5,67E+03 | 7 | 8 | 10 | 32359 Da |
| Shiga toxin subunit A | Cleaved StxA | Q9FBI2 | 60.54% | 100 % | 1,45E-03 | 13 | 23 | 27 | 32359 Da |
| Shiga toxin subunit A | StxA2a | Q9FBI2 | 3.40% | 83 % | 5,62E+02 | 0 | 0 | 1 | 32359 Da |
| Shiga toxin subunit A | Cleaved StxA2a | Q9FBI2 | 3.40% | 88 % | 5,70E+01 | 0 | 0 | 1 | 32359 Da |
| Shiga-like toxin 2 subunit A | StxA2a | P09385 | 20.38% | 100 % | 3,37E+03 | 5 | 5 | 6 | 35715 Da |
| Shiga-like toxin 2 subunit A | Cleaved StxA2a | P09385 | 22.57% | 100 % | 6,27E+02 | 7 | 10 | 11 | 35715 Da |

**Supplementary Table 2**: Identification parameters of mass spectrometry analysis from Scaffold-Analysis for StxA and StxA2a from *E. coli* -based cell-free synthesis.

| Protein | Sample | Accession | Coverage | Probability | Percentage of total Spectra | Exclusive unique peptides | Exclusive unique spectra count | Total spectra count | MW |
| --- | --- | --- | --- | --- | --- | --- | --- | --- | --- |
| Shiga toxin subunit A | NTC | Q9FBI2 | 0.37415 | 100 % | 3,56E+02 | 6 | 6 | 6 | 32359 Da |
| Shiga toxin subunit A | NTC potentially cleaved | Q9FBI2 | 0.472789 | 100 % | 7,00E+01 | 9 | 11 | 11 | 32359 Da |
| Shiga toxin subunit A | StxA | Q9FBI2 | 0.795918 | 100 % | 2,14E-03 | 19 | 32 | 34 | 32359 Da |
| Shiga toxin subunit A | Cleaved StxA | Q9FBI2 | 0.897959 | 100 % | 5,41E-03 | 34 | 60 | 90 | 32359 Da |
| Shiga toxin subunit A | StxA2a | Q9FBI2 | 0.091837 | 100 % | 2,45E+03 | 1 | 1 | 4 | 32359 Da |
| Shiga toxin subunit A | Cleaved StxA2a | Q9FBI2 | 0.139456 | 100 % | 1,32E+02 | 2 | 2 | 2 | 32359 Da |
| Shiga-like toxin 2 subunit A | StxA | P09385 | 0.413793 | 100 % | 5,66E+03 | 8 | 9 | 9 | 35715 Da |
| Shiga-like toxin 2 subunit A | Cleaved StxA | P09385 | 0.645768 | 100 % | 1,20E-03 | 12 | 15 | 20 | 35715 Da |
| Shiga-like toxin 2 subunit A | StxA2a | P09385 | 0.724138 | 100 % | 3,61E-03 | 31 | 44 | 59 | 35715 Da |
| Shiga-like toxin 2 subunit A | Cleaved StxA2a | P09385 | 0,76489 | 100 % | 2,04E-03 | 22 | 30 | 31 | 35715 Da |
